# Research progress of quercetin in cardiovascular disease

**DOI:** 10.3389/fcvm.2023.1203713

**Published:** 2023-11-16

**Authors:** Weiwei Zhang, Yan Zheng, Fang Yan, Mingqing Dong, Yazhou Ren

**Affiliations:** ^1^ Department of Oncology, Cancer Prevention and Treatment Institute of Chengdu, Chengdu Fifth People’s Hospital (The Second Clinical Medical College, Affiliated Fifth People’s Hospital of Chengdu University of Traditional Chinese Medicine), Chengdu, China; ^2^ School of Computer Science and Engineering, University of Electronic Science and Technology of China, Chengdu, China; ^3^ Geriatric Diseases Institute of Chengdu, Center for Medicine Research and Translation, Chengdu Fifth People’s Hospital, Chengdu, China

**Keywords:** quercetin, cardiovascular disease, antioxidant, lipid-lowering, myocardial protection

## Abstract

Quercetin is one of the most common flavonoids. More and more studies have found that quercetin has great potential utilization value in cardiovascular diseases (CVD), such as antioxidant, antiplatelet aggregation, antibacterial, cholesterol lowering, endothelial cell protection, etc. However, the medicinal value of quercetin is mostly limited to animal models and preclinical studies. Due to the complexity of the human body and functional structure compared to animals, more research is needed to explore whether quercetin has the same mechanism of action and pharmacological value as animal experiments. In order to systematically understand the clinical application value of quercetin, this article reviews the research progress of quercetin in CVD, including preclinical and clinical studies. We will focus on the relationship between quercetin and common CVD, such as atherosclerosis, myocardial infarction, ischemia reperfusion injury, heart failure, hypertension and arrhythmia, etc. By elaborating on the pathophysiological mechanism and clinical application research progress of quercetin's protective effect on CVD, data support is provided for the transformation of quercetin from laboratory to clinical application.

## 1. Introduction

Cardiovascular disease is a global chronic disease with high mortality and disability rates, and its pathogenic factors are complex and diverse, such as oxidative stress, inflammation, and arterial plaques (1, 2). Antioxidant, anti-inflammatory, and lipid-lowering treatment strategies are considered one of the treatment methods for preventing and treating CVD. Although Western medicine has a clear therapeutic effect on CVD, its target is relatively single. The research and development process of western medicine is slow and complex, requiring a significant investment of time, energy, and financial resources. Therefore, more and more researchers are paying attention to the therapeutic value of natural molecular compounds in CVD.

Quercetin, which has existed for a hundred years in the history of traditional Chinese medicine, belongs to a natural flavonoid compound. Quercetin is found in high concentrations in a variety of foods, including fruits, onion, tea, and red wine (3). As is well known, most of the chemotherapy drugs approved by the Food and Drug Administration (FDA) are extracted from natural products such as plants and marine organisms (4). As an important natural drug molecule, quercetin has been used alone or in combination for the treatment of various diseases, including malignant tumors, CVD, autoimmune diseases, metabolic diseases, etc (5–8). Human research on quercetin has never stopped, and by continuously improving its biological activity and bioavailability, more patients can benefit from it.

**Figure 1:**
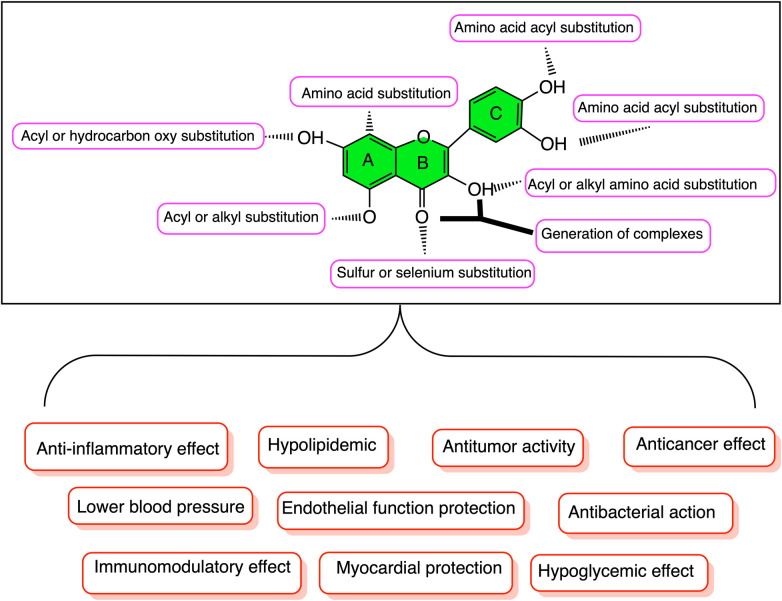
Structural modification and biological activity of quercetin.

Recently, a large number of *in vitro* and *in vivo* studies have shown that quercetin has various functions such as anti-inflammatory, antioxidant, antihypertensive, hypoglycemic, neurovascular protection, anticancer, anti-aging, and immune enhancement (7, 9–11) (Figure 1 and Table 1). Quercetin has prominent medicinal value in CVD, such as antioxidation, antiplatelet aggregation, reducing myocardial fibrosis, improving ventricular remodeling and cardiac function, protecting vascular endothelium, anti-arrhythmia, anti-heart failure, preventing ischemia reperfusion injury, and regulating blood pressure. This study will mainly describe the research progress of quercetin in CVD.

**Table 1:** Clinical trials of quercetin and CVD.

Diseases	Number of patients	Dosage regimen	Experiment Type	Primary outcomes	References
MI	88	500 mg/day orally for 8 weeks	Double blind, placebo-controlled, randomized clinical trial	↓Inflammatory factors, TAC; ↑QOL	(Dehghani et al.) (12)
Hypertension	22	162 mg/day orally for 6 weeks	Randomized, double-blind, placebo-controlled, crossover trial	BP and endothelial function→	(Brüll et al.) (13)
Hypertension	84	1,000 mg orally 2 times per day for 6 months, and then 500 mg 2 times per day for 6 months	Non randomized clinical trials	↑Left ventricular diastolic function, purine metabolism; ↓BP	(Kondratiuk et al.) (14)
Hypertension	41	730 mg/day orally for 28 days	Randomized, double-blind, placebo-controlled, crossover trial	↓BP; oxidative stress→	(Edwards et al.) (15)
Hypertension	18	25 mg/day orally for 28 days	Double-blinded, placebo-controlled, crossover trial	↓Diastolic pressure; ↑eNOS and NO	(Biesinger et al.) (16)
Hypertension	17	Total orally 1,095mg	Randomized, double-blind, cross-over, placebo-controlled study	↓BP; ACE and ET-1→	(Larson et al.) (17)
Hypertension	186	150 mg/day orally for 6 weeks	Double-blinded, placebo-controlled cross-over trial	↓BP; TNF-α, CRP→	(Egert et al.) (18)
High CVD risk phenotype	72	500 mg/day orally for 10 weeks	Double-blind randomized clinical trial	↓BP; LDL-C, TG, TNF-α, IL-6→	(Zahedi et al.) (19)
APOE genotype healthy male	49	150 mg/day orally for 8 weeks	Double-blind crossover study	↑TNF-α, waist circumference, postprandial systolic BP	(Pfeuffernet al.) (20)
Volunteers	22	30 mg/day orally for 2 weeks	Randomized, placebo controlled	↓ox-LDL	(Chopra et al.) (21)
Healthy volunteers	15	200 mg or 400 mg/day orally for 3 weeks	Double blind, randomized, placebo-controlled trial	Induce vasodilator effects	(Perez et al.) (22)
CHD	85	120 mg/day orally for 12 months	Randomized controlled trial	↑Left ventricular systolic and diastolic function, protect the heart	(Chekalina et al.) (23)

ACE, Angiotensin-converting enzyme; APOE, Apolipoprotein E; BP, Blood pressure; CHD, Coronary heart disease; CRP, C-reactive protein; CVD, Cardiovascular diseases; ET-1, Endothelin-1; eNOS, Endothelial nitricoxide synthase; IL-6, Interleukin-6; LDL-C, Low-density lipoprotein cholesterol; MI, Myocardial infarction; NO, Nitric oxide; ox-LDL, Oxidized low-density lipoprotein; QOL, Quality of life; TAC, Total antioxidant capacity; TAG, Triacylglycerol; TNF-α, Tumor necrosis factor α; TxA2, Thromboxane A2; TG, Triglycerides; ↑, Increase or increase, ↓, Down or down; →, Stable or no change.

## 2. Structure and metabolic pathway of quercetin

**Figure 2:**
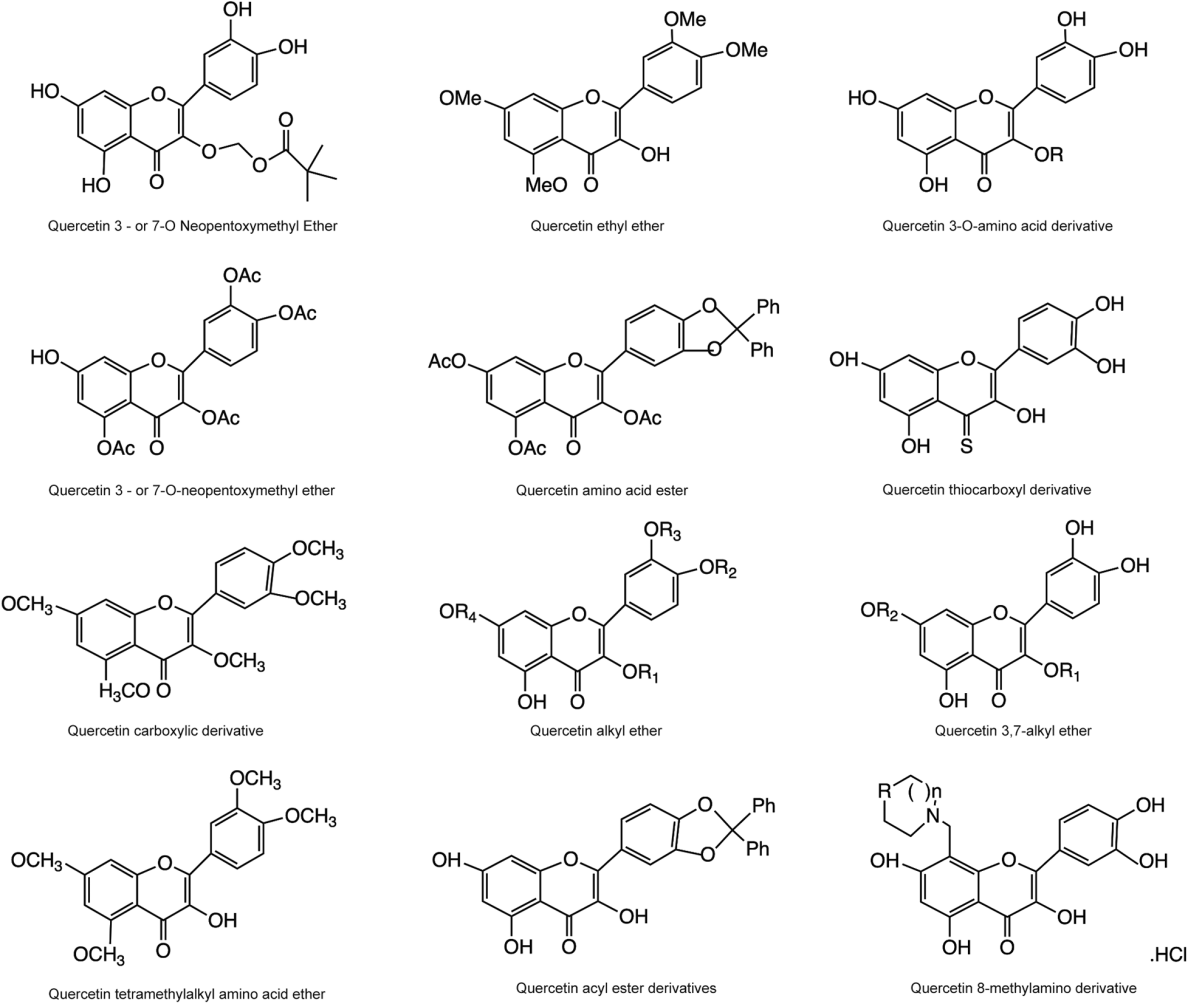
Structure of some derivatives of quercetin.

Quercetin has a structure of 3,3’, 4’, 5,7-pentahydroxyflavones, which are naturally present in the form of quercetin glycosides (24, 25). Quercetin is composed of two benzene rings (A ring and B ring) and a closed pyran ring (C-ring). The A ring has two hydroxyl groups that belong to the m-diphenol structure, the B ring has two hydroxyl groups that belong to the o-diphenol structure, and the C ring has one hydroxyl group that belongs to an enol structure, with a total of five hydroxyl groups (Figure 1). Its glycosylation can occur on any hydroxyl group, and by combining with glucose, xylose, or rutin sugar, various forms of quercetin glycosides are produced (26). As medical research continues to deepen, researchers have found that compounds obtained from natural products, such as quercetin, are more effective in treating and preventing diseases (27, 28). Although poor water solubility and low bioavailability limit the clinical application of quercetin, its metabolic derivatives can effectively clear the active substances in the body, making it considered by epidemiologists and nutritionists as a natural compound with the most promising application in disease prevention and treatment (29–31). The derivatives of quercetin mainly include O-glycosides, C-glycosides, ethers, and derivatives containing alkyl substituents (11, 25) (Figure 2).

**Figure 3:**
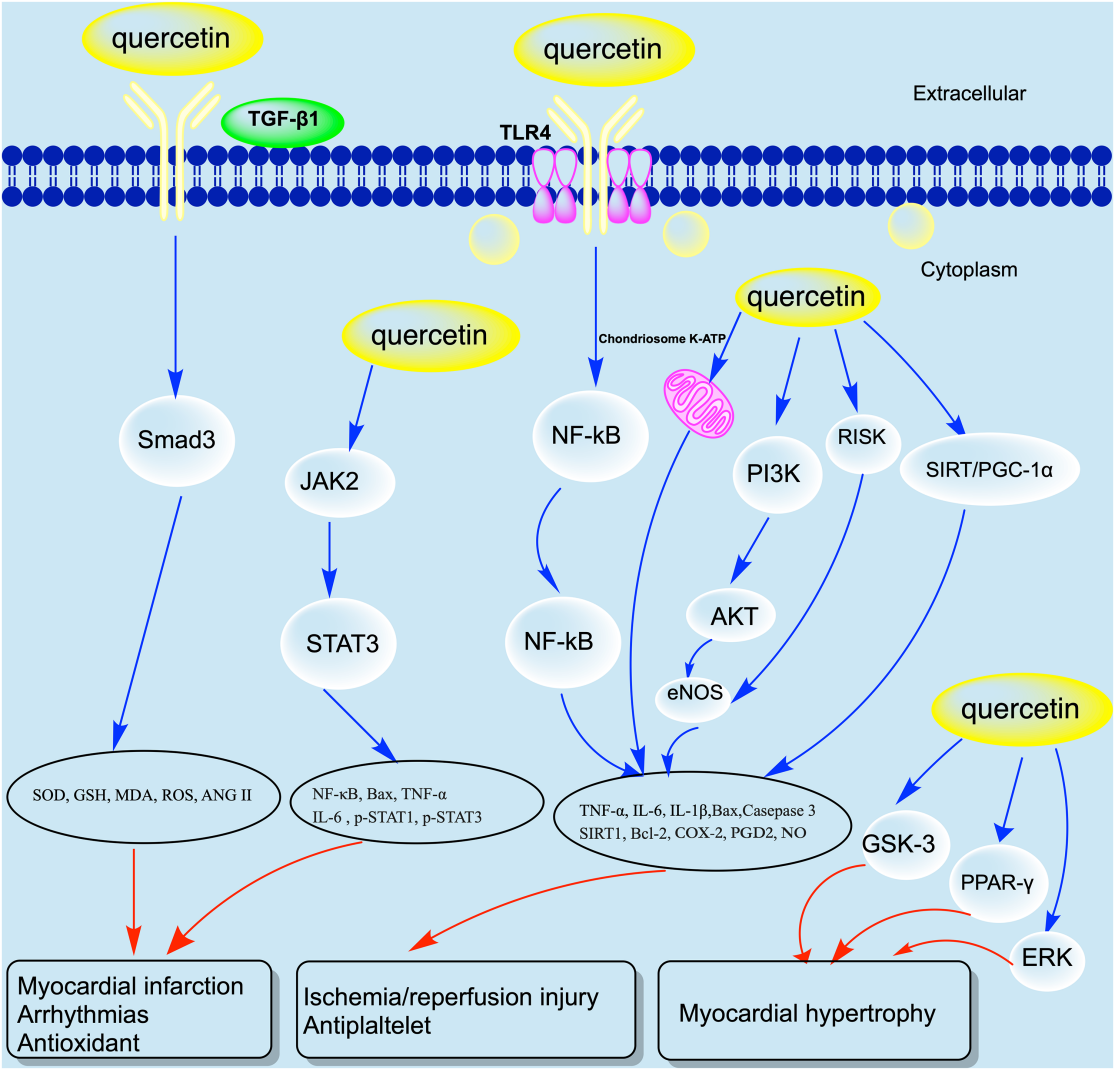
Molecular mechanism of quercetin in CVD. COX, cyclooxygenase; ERK, Extracellular regulated protein kinases; MDA, Malondialdehyde; NF-kB, Nuclear Factor Kappa Beta; NO, Nitric oxide; PDG2, Prostaglandin E2; PGC-1α, GSH, Glutathione; Peroxisome proliferator-activated receptor-*γ* Coactivator-1α; RISK, Reperfusion Injury Salvage Kinases; SOD, Superoxide dismutase; SIRT1, Silencing information regulator 1; STAT, Signal transducer and activator of transcription; TNF-α, Tumor necrosis factor α.

**Figure 4:**
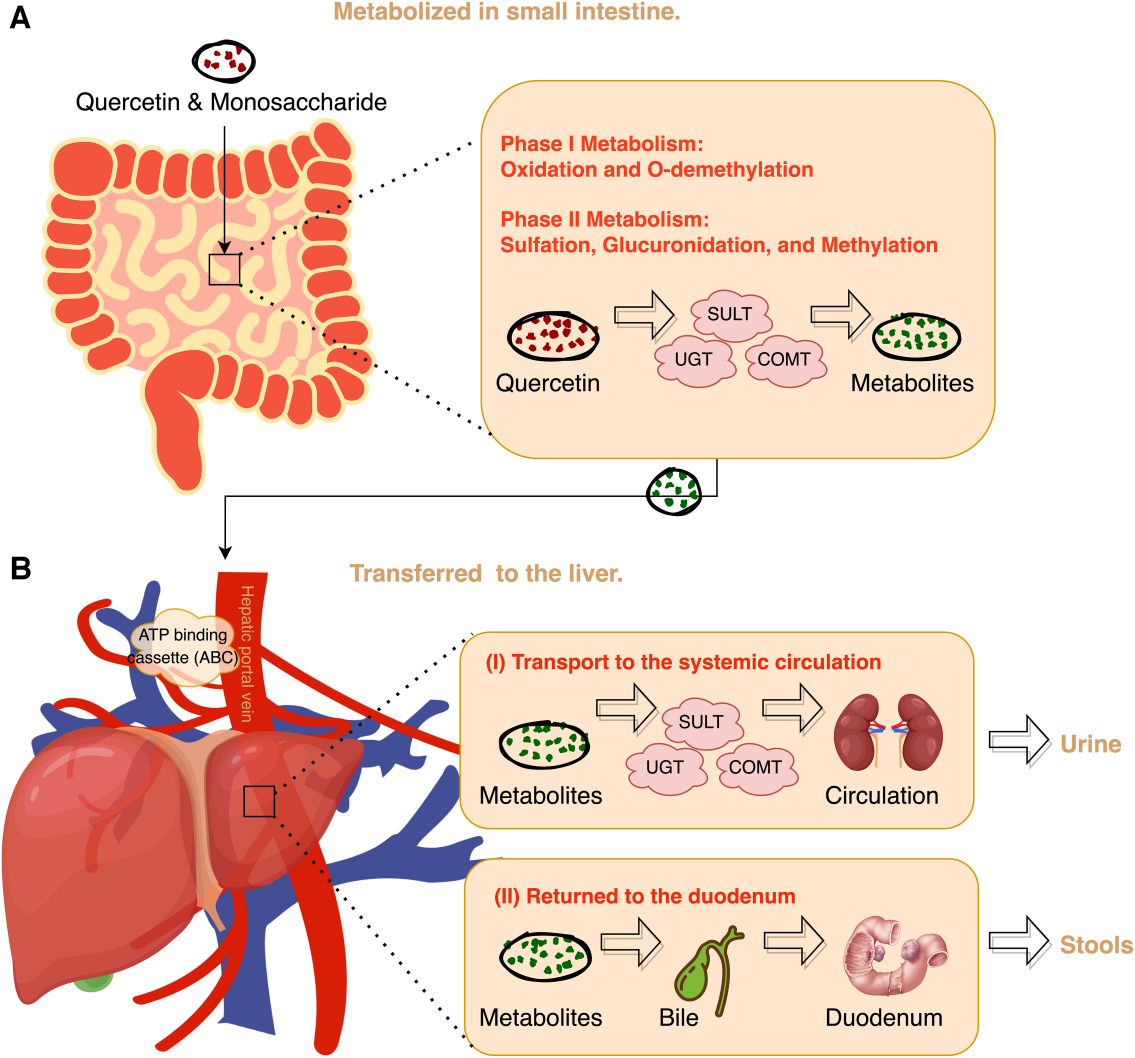
Overview of quercetin metabolization in the body. COMT, Catechol-O-methyltransferase; SULT, Sulfotransferase; UGT, UDP-glucuronosyltransferase.

Quercetin, as a relatively low molecular weight polyphenol compound, has broad pharmacological effects and therapeutic potential mainly through interactions with gut microbiota or key cell signaling proteins (32) (Figure 3). After oral administration, the quercetin is absorbed into the bloodstream by the small intestine in the form of glycosides, then bound to serum albumin and transported to the liver for metabolism (33, 34). Quercetin binds with methyl, sulfate, or glucuronic acid in the body to produce active metabolites such as isorhamnetin, kaempferol, and tamarind (35). These metabolites have a half-life of up to 28 h, and after being released, they reach the blood and lymph nodes of the whole body, and finally enter different organs such as the liver and kidneys for catabolism, which is ultimately discharged through feces or urine for 24 to 28 h (25, 36–40) (Figure 4).

## 3. The relationship between quercetin and CVD

### 3.1. Antioxidant effect

The antioxidant effect of quercetin is mainly achieved by directly clearing reactive oxygen species, chelating metal ions, and inhibiting LDL oxidative damage. Quercetin, as a natural antioxidant, can remove hydroxyl radicals, hydrogen peroxide (H_2_O_2_), and superoxide anions accumulated in cells both *in vivo* and *in vitro*, thereby increasing oxygen atoms to stabilize the structure of the benzene ring (41, 42). Quercetin can also induce the production of the antioxidant enzyme heme oxygenase-1 (HO-1), thereby enhancing cellular defense against oxidative damage (43). The antioxidant capacity of quercetin is mainly related to its ability to scavenge free radicals (44). We know that cellular oxidative stress damage caused by reactive oxygen species (ROS) is mainly related to signaling pathways such as nuclear factor carotenoid derivative 2 (NRF2), adenosine monophosphate activated protein kinase (AMPK), mitogen-activated protein kinase (MAPK), extracellular regulated protein kinases (ERK), p38, and c-Jun N-terminal kinase (JNK). Quercetin can just scavenge ROS, which is involved in maintaining intracellular oxidation balance. Zhang et al. (45) found that quercetin can clear ROS in cardiac fibroblasts and inhibit cell proliferation, which is related to inhibiting the activation of the MAPK signaling pathway to reduce the phosphorylation levels of ERK, p38, and JNK. Lu et al. (46) showed that quercetin can also activate caspase-3 and nuclear factor (NF) regulated by Phosphatidylinositol-3-kinase (PI3K)/ protein kinase κB pathway (Akt-κB) to reduce ROS production, thereby improving atherosclerosis.

In the oxidative stress model induced by hydrogen peroxide, quercetin pretreatment significantly reduced intracellular ROS levels (47). This indicates that quercetin can reduce intracellular reactive oxygen species and protect cells from oxidative damage. Researchers also found that quercetin significantly increased cell viability by reversing oxidative stress induced by hydrogen peroxide, while the expression levels of oxidative stress-related proteins induced by hydrogen peroxide also decreased. The direct scavenging effect of quercetin on reactive oxygen species may be related to the abundance of phenolic hydroxyl groups in the structure. Phenolic hydroxyl groups can exert antioxidant effects by providing active hydrogen to inactivate free radicals while being oxidized to highly stable free radicals (48).

In addition, antioxidant enzymes such as catalase (CAT) and superoxide dismutase (SOD) play an important role in clearing superoxide anion free radicals in the body. A study found that quercetin can protect the myocardium from damage by increasing the activity of antioxidant enzymes in rats with acute myocardial infarction, including SOD, catalase and gluthation peroxidase (49). Quercetin can significantly reduce the levels of oxidative stress biomarkers such as malondialdehyde (MDA) and nitric oxide synthase (iNOS) in hypoxic induced myocardial tissue of rats, while increasing the activity of SOD and CAT. In a cadmium induced cardiovascular disease rat model, quercetin can also protect the heart by increasing SOD, CAT, and glutathione peroxidase (50).

The steady state destruction of metal iron and copper in the body is also one of the reasons for the increase of free radicals in the body (51). Quercetin has a strong ability to chelate metal ions, thereby blocking the Fenton reaction and ROS production (52). In stable chelating complexes, quercetin exhibits stronger antioxidant effects. Research has found that catechol in the molecular structure of quercetin can chelate with Cu^2+^ and Fe^2+^ to exert antioxidant effect (53). In the model of alcoholic liver disease, quercetin inhibits Fe^2+^ induced lipid peroxidation by chelating Fe^2+^, ultimately inhibiting iron overload and oxidative damage caused by alcoholic liver disease (54). Pękal et al. (55) found through spectral analysis that under the action of Cu^2+^, quercetin can be oxidized into benzoquinone products with stable structures, and Cu^2+^ also loses its ability to mediate lipid oxidation. In addition, Jomova et al. (56) found that the chelation of quercetin with copper can significantly inhibit the ability of copper to induce hydroxyl radical formation, and quercetin can also protect DNA from reactive oxygen species by inhibiting the formation of reactive oxygen species.

The increase of oxidized low-density lipoprotein (ox-LDL) *in vivo* will not only lead to the necrosis or apoptosis of vascular endothelial cells, inflammatory cells, fibroblasts and smooth muscle cells, but also promote the development of atherosclerosis and other CVD (57–59). Quercetin can achieve antioxidant effects by inhibiting LDL oxidation and reducing intracellular ROS content (60). Hertog et al. (61) observed that when plasma levels of quercetin increase, both total cholesterol and low-density lipoprotein cholesterol levels decrease. This may be related to quercetin upregulating MAPK and ERK phosphorylation to promote autophagy, thereby promoting cell survival. In addition, quercetin inhibits ox-LDL induced oxidative stress by downregulating the expression of toll like receptor 4 (TLR4) in the ROS/TLR4 signaling pathway, thereby reducing ox-LDL induced cell calcification and osteogenic differentiation of vascular smooth muscle cells (62).

### 3.2. Anti atherosclerotic effect

Atherosclerosis is a chronic vascular inflammatory disease related to ox-LDL (63). With the continuous stimulation of inflammatory factors in the arterial wall and the accumulation of lipids in the intima, vascular endothelial cells can be damaged, leading to dysfunction (64). Although the use of anti-lipid drugs has certain preventive and therapeutic effects on atherosclerosis, the incidence of cardiovascular events is still high. In recent years, researchers have found that combined use of anti-inflammatory drugs can effectively alleviate and treat arterial Congee. Reducing blood lipids and cholesterol in atherosclerotic plaques is an important treatment to inhibit the progression of atherosclerosis.

As a purely natural drug, quercetin has a strong anti-inflammatory effect, mainly inhibiting inflammatory factors such as interleukin (IL) - 6, IL-1β, monocyte chemotactic protein 1 (MCP-1), and vascular endothelial growth factor (VEGF) (65). Quercetin can activate caspase-3 and NFK-β factor and paraoxonase 1 gene expression (46), and inhibition of endoplasmic reticulum stress chop pathway to inhibit the development of atherosclerosis (66). In addition, quercetin can also inhibit the release of inflammatory factors in macrophages. Zhang et al. (67) found that quercetin can reduce the inflammatory factor IL-1 in a rat model of cerebral ischemia caused by IL-1β and IL-6 to alleviate the severity of cerebral ischemia. Si et al. (68) found that quercetin can regulate Nuclear Factor Kappa Beta (NF-κB) and MAPK signaling pathways inhibit the secretion of prostaglandin E2 (PGD2), cyclooxygenase-2 (COX-2), and nitric oxide (NO). It was also found in animal models that quercetin could reduce the expression levels of matrix metallopeptidase (MMP)-1 and MMP-9 by inhibiting ERK signaling pathway, thus stabilizing atherosclerotic plaque (69, 70).

In the ApoE knockout mouse model, quercetin can significantly reduce the atherosclerotic plaque area in the hyperlipidemia group, and alleviate the oxidative stress response of various systems by blocking the activation of Nicotinamide adenine dinucleotide phosphate (NADPH) oxidase (71). In addition, quercetin can significantly increase the activity of nitric oxide synthase and the expression of HO-1 protein in endothelial cells (72). In cell and animal models of atherosclerosis induced by high fructose or lipopolysaccharide, quercetin indirectly affects PI3K/Akt pathway mainly by regulating ROS, thereby inhibiting the occurrence of inflammation and apoptosis, and ultimately reducing the degree of atherosclerosis (46). In other mouse models, exercise and quercetin can reduce the formation of atherosclerotic plaques by 78% (73), which may be related to the effect of quercetin on blood lipid (74).

### 3.3. Myocardial infarction

Myocardial infarction (MI) is a heart disease with high mortality and disability rates caused by the blockage of blood in a certain part of the heart, leading to the death of myocardial tissue (75). MI can cause systemic and local inflammatory reactions in the body, increasing the release of inflammatory factors, which in turn exacerbates further damage to the myocardium. Ischemic myocardial tissue can recruit a large number of neutrophils, thereby increasing the production of ROS (12). Myocardial fibrosis and ventricular remodeling are common pathological changes in the late stages of MI (76–78). The quality of life of patients with MI has also significantly decreased. In addition to existing medication, many traditional Chinese medicine health foods are also recommended for adjuvant treatment of MI (79).

Angiotensin II (Ang II) is an important fibrogenic factor leading to myocardial fibrosis. Some studies have found that quercetin can reduce the effects of Ang II on myocardial fibrosis and hypertrophy, and reverse mouse ventricular remodeling (80, 81). In the rat model of ventricular hypertrophy, quercetin was found to be responsible for the inhibition of Silencing information regulator 1 (SIRT1)/NF-κB pathway alleviates left ventricular hypertrophy in rats (82, 83). Lacerda et al. (84) also found that quercetin can reduce ROS production by increasing the content of endogenous antioxidants in mice, thereby reversing myocardial hypertrophy and improving cardiac function. In the rat heart failure model, quercetin was also found to improve cardiac function by increasing the expression of NRF2 to restore reconstructed cardiomyocytes (85). The transforming growth factor (TGF) β1 superfamily is also an important factor that causes myocardial fibrosis and apoptosis after MI. In another preclinical study on rats with MI, it was found that quercetin can inhibit the TGFβ1/Smad3 signaling pathway to eliminate ROS and enhance the myocardial antioxidant, anti-inflammatory, and anti-fibrosis capabilities, thereby reversing ventricular remodeling (86).

In addition, Janus kinase (JAK)/signal converter and transcription activating factor (STAT) signaling pathways may also be involved in the protective effect of quercetin on MI. Oral administration of 50 mg/kg quercetin in rats with acute MI can significantly increase the expression levels of IL-6, Bax, NF-kB p65, tumor necrosis factor α (TNF-a) through the JAK/STAT signaling pathway, while reducing the expression of p-STAT1 (Ser727) (87). Li et al. (49) also found that quercetin has a protective effect on acute MI in both low and high dose groups and can significantly reduce TNF-α and IL-1β while increasing antioxidant capacity.

Although quercetin has achieved promising results at both the cellular and animal levels, its potential benefits in preventing and managing human MI have not been well confirmed. While there have been a few small human studies exploring the effects of quercetin supplementation on heart health, the results have been inconsistent, and more well-designed clinical trials are needed to fully evaluate the potential benefits of this polyphenol. A double-blind, placebo-controlled, randomized clinical trial showed that after 8 weeks of oral treatment with 500 mg quercetin or placebo in 88 MI patients, the serum total antioxidant capacity (TAC) of the quercetin group was significantly improved, while the inflammatory factor TNF-α was also significantly reduced (12). However, there were no significant changes in IL-6, C-reactive protein (CRP), and blood pressure in both groups of patients (12). Lu et al. (88) demonstrated that onion juice containing quercetin can significantly inhibit the regulation of lipid status and antioxidant status in patients with mild hypercholesterolemia. However, some studies have shown that quercetin has no significant impact on serum TAC, TNF-α, and IL-6 levels in patients (89–91). This may be related to the varying nature of the disease. Overall, there are currently very few clinical trials of quercetin for MI. It can be seen that more carefully designed clinical trials are needed to further explore the protective effects and mechanisms of quercetin on MI (Table 2).

**Table 2:** Preclinical studies of quercetin and MI.

Animal	Study design	Signal pathway	Outcomes	References
Rat	50 mg/kg orally for 30 days	TGF-β1/Smad3	IL-6 and TNF-α→; ↓SOD, GSH, MDA, ROS and ANG II	(Albadrani et al.) (86)
Rat	50 mg/kg orally for 7 days	JAK2/STAT3	↓ NF-κB, Bax, TNF-α; ↑IL-6, p-STAT1 and p-STAT3	(Albadrani et al.) (87)
Rat	Total 100 mg/kg or 400 mg/kg gavage	/	↓TNF-α, IL-1β, MDA, SOD and CAT	(Li et al.) (49)
Rat	5 mg/kg or 10 mg/kg i.p 10 min before reperfusion	/	↑SOD and CAT	(Annapurna et al.) (92)

ANG II, Angiotensin II; CAT, Catalase; i.p, Intraperitoneal perfusion; IL-6, Interleukin-6; IL-1β, Interleukin-1β; GSH, Glutathione; JAK2/STAT3, Janus kinase signal transducers 2 and activator of transcription 3; MDA, Malondialdehyde; MI, Myocardial infarction; p-STAT, phosphorylated Signal Transducer And Activator Of Transcription; ROS, Reactive oxygen species; SOD, Superoxide dismutase; NF-kB, Nuclear Factor Kappa Beta; TGF-β1, Transforming growth factor β1, TNF-α, Tumor necrosis factor α; ↑, Increase or increase, ↓, Down or down; →, Stable or no change.

### 3.4. Ischemia/reperfusion injury

As one of the main risk factors for coronary heart disease (CHD), ischemia and reperfusion injury (I/R) can produce a large amount of ROS, leading to myocardial cell death, arrhythmia, and dysfunction (93, 94). Therefore, protecting ischemic myocardium is crucial in the treatment of CHD and angina pectoris. As a polyphenolic compound, quercetin has been proven to have protective effects on a variety of cells in I/R, such as myocardial cells, liver cells, and kidney cells (44).

However, the mechanism by which quercetin protects myocardial cells from I/R is not fully understood. Sanhueza et al. (95) found that quercetin can prevent the decrease of the xanthine dehydrogenase/xanthine oxidase ratio, thereby reducing the oxidative damage caused by I/R in myocardial cells. Chen et al. (96) found that quercetin protects cardiomyocytes by reducing Src kinase, STAT3, caspase 9, Bax, intracellular reactive oxidation products, and inflammatory factors. In a study where young rats (4 weeks) and adult rats (12 weeks) were treated with quercetin at a dose of 20 mg/kg per day for 4 weeks, the isolated hearts were subjected to ischemia for 25 min and then reperfusion for 40 min. The results showed that quercetin improved left ventricular end-diastolic pressure in young rats after ischemia but had no effect on adult rats (97). This suggests that the dose and duration of quercetin use in CVD should consider age as a factor.

Similarly, in another isolated I/R injury model, it was found that quercetin can improve myocardial injury through the high mobility group box 1 (HMGB1) pathway (98). The addition of quercetin to the myocardial ischemia-reperfusion solution of male Wistar rats significantly reduce IL-1β, TNF-α and IL-6 level through mitochondrial adenosine triphosphate (ATP) sensitive potassium channels and NO systems (99). In an I/R model induced by coronary artery occlusion for 30 min and reperfusion for 2 h, quercetin significantly reduced the MI area, inhibited cardiomyocyte apoptosis and caspase-3 immune response, and decreased serum creatine kinase and lactate dehydrogenase levels (100). In addition, quercetin can also increase Akt phosphorylation and Bcl-2 expression through the PI3K/Akt signaling pathway, as well as reduce Bax expression (101). This result has also been further confirmed by Liu's study (101). There are also studies using quercetin (1.0 mg/kg, i.v.) to treat isolated rat heart *I*/*R* injury, and the results showed that serum TNF-α and IL-10 expression decreased significantly (102). In addition, quercetin in combination with amlodipine can increase cardiac function, ATP, and reduced glutathione (GSH) levels while reducing the levels of creatine kinase (CK), thiobarbituric acid reactive substances (TBARS) and total nitrate/nitrite (x) (103). Wan et al. (104) also found that quercetin can reduce the levels of Nitrogen oxide compound (NOX) and nitrous oxide system (NOS) proteins and mRNA to protect against myocardial injury in the I/R rabbit model. Brookes et al. (105) found that taking quercetin (0.033 mg/kg per day, gavage for 4 days) in rats can stabilize mitochondrial function and protect myocardial *I*/*R*.

However, in the *I*/*R* model of type 2 diabetes rats, although quercetin upregulates the expression of endothelial nitricoxide synthase (eNOS) in young rats and protein Kinase C (PKC) Epsilon in old rats, it does not activate the entire PI3K/Akt pathway (23). Therefore, it has not shown any cardiac protective effect, and even worsened the cardiac function of rats over 1 year old. It can be seen that although quercetin has shown good myocardial protection in *I*/*R* animal models, it still needs further verification in *I*/*R* animal models with other diseases such as diabetes.

Although there is sufficient evidence in preclinical studies of quercetin in the treatment of simple *I*/*R* diseases, there are still few studies actually used in clinical trials. In a study comparing quercetin and aspirin in the treatment of patients with myocardial ischemia, it was found that after 2 months of treatment with 120 mg/kg quercetin, cardiac function, hemodynamics, and symptoms of myocardial ischemia were significantly improved (106). In addition, among 55 patients with chronic ischemic heart disease with metabolic syndrome, 35 patients receiving quercetin treatment significantly reduced the incidence and duration of myocardial ischemia, reduced supraventricular extrasystole to 5%, and significantly reduced the incidence of arrhythmia (107) (Table 3).

**Table 3:** Preclinical studies of quercetin and I/R.

Animal	Study design	Signal pathway	Outcomes	References
Rat	50 mg/kg orally for 5 days	HMGB1-TLR4-NF-κB	↓TNF-α, IL-6 and IL-1β	(Dong et al.) (98)
Rat	100 ng/ml cardiac perfusion for 10 min	NO, Mitochondrial K-ATP Channels	↓IL-1β, TNF-α, IL-6	(Liu et al.) (99)
Rat	40 μmol/L for 10 days	JAK2/STAT3	↓Apoptosis and oxidative stress, MI area; ↑Ventricular remodeling and biochemical indicators, recovery of cardiac blood flow	(Liu et al.) (108)
Rat	10 mg/kg *ip* 5 min before reperfusion	PI3K/Akt	↓MI area, cardiomyocyte apoptosis and caspase-3, CK, LDH and Bax; ↑Akt phosphorylation and Bcl-2	(Wang et al.) (100)
Rat	20 mg/kg orally for 6 weeks	RISK, NO	↑eNOS in younger rats, ↑PKC*ε* in older rats, did not activate PI3K/Akt pathway	(Ferenczyova et al.) (23)
Rat	1.0 mg/kg i.v. once total	/	↓TNF-α, IL-10	(Jin et al.) (102)
Rat	25 mg/kg or 50 mg/kg or 100 mg/kg by gavage for 1 weeks before operation	SIRT1/PGC-1α	↑SIRT1, PGC-1α, Bcl-2; ↓Bax	(Tang et al.) (109)
Rat	20 mg/kg orally for 4 weeks	/	↓Left ventricular end-diastolic pressure	(Bartekova et al.) (97)
Rat	250 mg/kg orally for 10 days	PI3K/Akt	↓TNF-α, CRP, IL-1β, Bax, ↑Bcl-2	(Liu et al.) (101)
Rat	5 mg/kg orally for 1 week before the operation	/	↑Cardiac function, ATP, and GSH; ↓CK, TBARS and nitrate/nitrite (x)	(Ahmed et al.) (103)
Rabbit	1 mg/kg i.v. 5 min before ligation, once total	NOX and NOS	↓NOX2, eNOS, iNOS	(Wan et al.) (104)

ATP, Adenosine triphosphate; Akt, protein kinase κB pathway; Bcl-2, B-cell lymphoma-2; CK, Creatine kinase; CRP, C-reactive protein; eNOS, endothelial nitricoxide synthase; HF, Heart failure;HMGB1, High mobility group box 1; i.v, Injected intravenously; i.p, Intraperitoneal perfusion; IL-6, Interleukin-6; IL-1β, Interleukin-1β; JAK2/STAT3, Janus kinase signal transducers 2 and activator of transcription 3; LDH, Lactate dehydrogenase; MI, Myocardial infarction; MDA, Malondialdehyde; NF-kB, Nuclear Factor Kappa Beta; NO, Nitric oxide; NOS, nitrous oxide system; NOX, Nitrogen oxide compound; PI3K, Phosphatidylinositol-3-kinase; PGC-1α, Peroxisome proliferator-activated receptor- γ Coactivator - 1α; PKCε, Protein Kinase C Epsilon; iNOS, inducible nitric oxide synthase; SIRT1, Silencing information regulator 1; TBARS, Thiobarbituric acid reactive substances; TLR4, Toll like receptor 4; TNF-α, Tumor necrosis factor α; VLDL, Very low-density lipoprotein; ↑, Increase or increase, ↓, Down or down; →, Stable or no change.

### 3.5. Myocardial hypertrophy

Myocardial hypertrophy is a compensatory response to increased stress in the myocardial wall, but as pressure continues to increase, it gradually becomes decompensated, leading to heart failure. Reversing myocardial hypertrophy is an important treatment for preventing heart failure. Cardiac hypertrophy is associated with many signal pathways and gene expression, including MAPK, JAK/STAT, and Activator protein-1 (AP-1) (110, 111). Ultimately, it leads to an increase in the concentration of Ca^2+^ in myocardial cells to promote myocardial hypertrophy. Quercetin can prevent myocardial hypertrophy by reducing the oscillation frequency of Ca^2+^ in rat cardiomyocytes (112).

In rats with constricted abdominal aorta, adding 1.5 g/kg of quercetin to their diet can lower blood pressure and reduce myocardial hypertrophy (113). Both *in vivo* and *in vitro* experiments have confirmed that quercetin can inhibit Ang II induced myocardial hypertrophy by enhancing PPAR-1 expression and inhibiting AP-1 activity (110). Similarly, quercetin can also inhibit Ang II induced myocardial hypertrophy through PKC and tyrosine protein kinase (TPK) signaling pathways (114). Han et al. (115) demonstrated that quercetin can inhibit the cardiac hypertrophy by inhibiting the ERK1/2, p38 MAP kinase, Akt and GSK-3betaβ activities in pressure overload rats.

Hypercholesterolemia is another risk factor for hypertrophic cardiomyopathy. In Apo E knockout mice, continuous oral administration of 0.1 µ mol/kg quercetin for 6 weeks significantly reduced total cholesterol and very low-density lipoprotein (VLDL) in peripheral blood, thereby inhibiting ventricular hypertrophy (116). Quercetin can also inhibit cardiomyocyte hypertrophy and apoptosis in rats through the NOX2/GAPDH pathway (116). Although these preclinical studies confirm that quercetin can inhibit the development of ventricular hypertrophy, unfortunately, there are currently no relevant clinical trials to confirm its role in patients with ventricular hypertrophy. We hope there will be real event studies on the prevention and treatment of myocardial hypertrophy diseases with quercetin in the future (Table 4).

**Table 4:** Preclinical studies of quercetin and myocardial hypertrophy.

Animal	Study design	Signal pathway	Outcomes	References
Rat	5 mg/kg or 10 mg/kg or 20 mg/kg orally for 8 weeks	GSK-3 Pathway	↓AKT, LKB1/AMPKα, ERK, histone H3, β-catenin, and GATA4	(Chen et al.) (117)
Rat	5 mg/kg or 10 mg/kg by gavage for 12 weeks	PPAR-*γ* and AP-1	↓Cardiac hypertrophy	(Yan et al.) (110)
Rat	In the drinking water at 5 mg or 10 mg /head for 3 weeks	ERK1/2, p38 MAP kinase, Akt and GSK-3b	↓Cardiac hypertrophy	(Han et al.) (115)
Rat	120 mg/kg i.v. for 5 days	Myocardial [Ca2+] i-oscillation	↓Heart rate, hypertrophy	(Wang et al.) (112)
Rat	0.1 µ mol/kg oral for 6 weeks	/	↓Cholesterol and VLDL	(Ulasova et al.) (116)
Rat	5 mg/kg by gavage for 3 weeks	Nox2/GAPDH	↓NADPH oxidase gene, myocardial hypertrophy and apoptosis	(Mao et al.) (116)

Akt, Serine/threonine kinase; AMPKα, Adenosine monophosphate activated protein kinase α; AP-1, Activator protein-1; ERK, Extracellular regulated protein kinases; GAPDH, Glyceraldehyde-3-phosphate dehydrogenase; GSK-3, Hepatose Synthase Kinase 3; i.v, Injected intravenously; PPAR-γ, Peroxisome proliferator-activated receptor γ; LKB1, Liver kinase B1; MAP, Mitogen-activated protein; NADPH, Nicotinamide adenine dinucleotide phosphate; NOX, Nitrogen oxide compound; VLDL, Very low-density lipoprotein; ↑, Increase or increase, ↓, Down or down; →, Stable or no change.

### 3.6. Hypertension

With the acceleration of the global population aging, the prevalence of hypertension in developing countries is also increasing year by year, and most of them are hypertension of unknown etiology (118). Hypertension is a chronic disease associated with endothelial dysfunction, smooth muscle cell contraction, and hyperlipidemia. Over time, hypertension can lead to various complications such as heart failure, myocardial hypertrophy, stroke, and CHD (119). Although there are many clinical options for antihypertensive drugs, some patients still have poor antihypertensive effects, and even develop refractory hypertension. Therefore, drug update and development in the treatment of hypertension is particularly important. In recent years, studies have found that quercetin has a unique pharmacological activity in reducing blood pressure.

Kim et al. (120) found that quercetin can inhibit the contraction of vascular smooth muscle through AMPK signaling pathway, thereby playing a role in reducing blood pressure. Lin et al. (121) have found that quercetin has a hypotensive effect by promoting autophagy of endothelial cells. Pereira et al. (122) found that quercetin can improve vascular remodeling and endothelial oxidative stress, thereby reducing systolic blood pressure. In a rat model of hypertension induced by renin angiotensin aldosterone (RAAS), quercetin can reduce blood pressure by increasing urine and promoting sodium excretion (123). In spontaneously hypertensive rats, continuous oral administration of quercetin (10 mg/kg) for 5 weeks significantly reduced blood pressure and malondialdehyde levels, and increased glutathione peroxidase activity (124, 125). In addition, quercetin also has a hypotensive effect in pregnancy induced hypertension, which may be related to the regulation of endothelin 1 (ET-1) and endothelin 1A receptor (ETAR) (126).

In the treatment of hypertension, quercetin can reduce hypertension induced aortic remodeling, oxidative stress, and MMP-2 activity (122). Quercetin can also reduce systolic and diastolic blood pressure in rats by reducing oxidative stress and NF-κB (127, 128). In a sodium fluoride induced hypertension model, quercetin can reduce blood pressure in rats by regulating the hsp70/ERK/PPAR pathway (129). Quercetin can reduce the activity of NADPH oxidase and vascular superoxide in hypertensive rat models, thereby improving vascular endothelial function and lowering blood pressure (13, 14). Even in sodium chloride induced hypertension, quercetin is superior to nifedipine in improving hemodynamics, redox, and metabolic imbalances (15).

In a clinical study of quercetin in the treatment of grade 2 hypertension, it was found that quercetin can significantly reduce the levels of nitric oxide, CRP, IL-1, and lipid profile in patients' peripheral blood (16). Adding quercetin to the treatment regimen for patients with hypertension and gout can improve left ventricular diastolic function, purine metabolism, and lower blood pressure (17). However, in a randomized, double-blind, controlled, crossover dietary study, adding 162 mg of quercetin to the diet per day did not improve blood pressure in hypertensive patients (18). In addition, in 93 overweight or obese individuals, quercetin reduced blood pressure in overweight subjects, but had no effect on TNF-α and C-reactive protein (19). Although quercetin can reduce systolic blood pressure in patients, it has no significant effects on other cardiovascular risk factors such as cholesterol, low density lipoprotein cholesterol (LDL-C), triglycerides, TNF-α and IL-6 (20). But the results of another randomized clinical trial were exactly the opposite. The waist circumference, triacylglycerol and postprandial systolic blood pressure of healthy men with apolipoprotein E (APOE) genotype significantly decreased after oral administration of quercetin, while the level of TNF-α and high-density lipoprotein cholesterol (HDL-C) significantly increased (21).

Another randomized, double-blind, crossover clinical study found that 41 hypertensive patients had a significant decrease in blood pressure after 28 days of continuous administration of 730 mg quercetin, while the measured oxidative stress index in plasma and urine remained unchanged, which was contrary to previous animal experimental studies (22). Similarly, another study also found that although quercetin can reduce blood pressure, it has nothing to do with angiotensin converting enzyme (ACE) activity and ET-1 (130). Therefore, the mechanism of quercetin in reducing hypertension still needs further in-depth research and exploration (Table 5).

**Table 5:** Preclinical studies of quercetin and hypertension.

Animal	Study design	Signal pathway	Outcomes	References
Rat	10 mg/kg orally for 6 weeks	Endothelial autophagy	↓BP	(Lin et al.) (121)
Rat	10 mg/kg i.p. for 4 weeks	RAAS	↓BP	(Mackraj et al.) (123)
Rat	10 mg/kg orally for 5 weeks	/	↓BP and malondialdehyde; ↑glutathione peroxidase activity	(Duate et al.) (124, 125)
Rat	10 mg/kg or 20 mg/kg or 50 mg/kg by gavage for 5 days	ET-1, sFlt-1	↓BP	(Sun et al.) (126)
Rat	10 mg/kg by gavage for 3 weeks	Oxidative stress	↓Vascular remodeling, oxidative stress and MMP-2 activity	(Pereira et al.) (122)
Rat	10 mg/kg by gavage for 2 weeks	NF-kB	↓Systolic and diastolic BP	(Ajibade et al.) (127)
Rat	50 mg/kg or 100 mg/kg by gavage for 1 week	HSP 70/ERK/PPARγ	↓BP	(Oyagbemi et al.) (129)
Rat	50 mg/kg by gavage for 3 weeks	Nitrite and nitroso	↓NADPH oxidase activity and vascular superoxide production	(Montenegro et al.) (13)
Rat	10 mg/kg orally by 13 weeks	↓NADPH oxidase, ↑ eNOS activity	↓BP	(Sánchez et al.) (14)

BP, Blood pressure; eNOS, endothelial nitricoxide synthase; ET-1, Endothelin-1; ERK, Extracellular regulated protein kinases; HSP, Heat Shock Protein; i.p, Intraperitoneal perfusion; MMP-2, Matrix metallopeptidase; NF-kB, Nuclear Factor Kappa Beta; PPAR-γ, Peroxisome proliferator-activated receptor γ; RAAS, Reninangiotensin-aldosterone; sFlt-1, Soluble fms-like tyrosine kinase-1; ↑, Increase or increase, ↓, Down or down; →, Stable or no change.

### 3.7. Heart failure

Heart failure (HF) is one of the serious CVD in clinical practice, and the main therapeutic drugs are β Receptor blockers, angiotensin converting enzyme inhibitors, or Ang II receptor antagonists. HF is closely related to cardiac hypertrophy and oxidative stress caused by ROS. Many studies have confirmed that quercetin, a ROS scavenger, can improve redox balance and mitochondrial homeostasis by blocking H_2_O_2_ and reversing mitochondrial Mn SOD activity, thereby reducing myocardial hypertrophy (84). Tan et al. (131) analyzed the network pharmacology system and found that quercetin may further improve the pathophysiological changes of HF by regulating the AKT1-eNOS-MMP9 pathway to resist apoptosis. In an *in vitro* experiment of cisplatin induced cardiac toxicity, it was found that H9c2 cardiomyocytes treated with 40 µM quercetin significantly decreased their myocardial cytotoxicity, which may be related to the Nrf2/HO-1 and P38MAKP/NF-κBp65/IL-8 signal pathway (132). Furthermore, quercetin can prevent myocardial hypertrophy through proteasome GSK-3 Pathway, which may be related to upstream liver kinase B1/AMP activated protein kinase (LKB1/AMPK α), protein kinase B and downstream hypertrophy factors such as extracellularly ERK, histone H3, β-Catenin, and GATA binding protein 4 (GATA4) (133). In a mouse model of HF, quercetin promotes the de succinylation of isocitrate dehydrogenase (IDH2) through SIRT5, maintains mitochondrial homeostasis, and improves myocardial fibrosis, thereby reducing the incidence of HF (134). Although there are currently no human experimental studies on the correlation between quercetin and HF, we believe there will be breakthroughs in the near future (Table 6).

**Table 6:** Preclinical study of quercetin and HF, AF, CHD and hyperlipidemia.

Animal	Diseases	Study design	Signal pathway	Outcomes	References
Rat	HF	50 mg/kg i.p for 4 weeks	SIRT5	↑IDH2, ↓HF	(Chang et al.) (134)
Rat	AF	25 mg/kg by gavage for 3 weeks	TGF-β/Smads pathway	↓miR-135b, ↓AF	(Wang et al.) (135)
Rat	AF	25 mg/kg by gavage for 3 weeks	miR-223-3p/FOXO3	↑Autophagy	(Hu et al.) (136)
Rat	CHD	50 mg/kg or 100 mg/kg or 200 mg/kg/times i.v or i.p, 3 times/week for 2 weeks	HMG-CoA	↓HMG-CoA	(Khamis et al.) (137)
Rat	Hyperlipidemia	4.0 g/kg supplement diet orally for 5 weeks	CYP7A1	Promote cholesterol-to-bile acid conversion	(Zhang et al.) (138)

AF, Atrial fibrillation; CHD, Coronary heart disease; CYP7A1, Cytochrome P450 7A1 protein; FOXO3, Fork head Box Protein O3; HF, Heart failure; HMG-CoA, 3-hydroxy-3-methyl glutaryl coenzyme A reductase; IDH, isocitrate dehydrogenase; i.p, Intraperitoneal perfusion; i.v, Injected intravenously; RISK, Reperfusion injury salvage kinases; SIRT5, Sirtuin 5; SOD, Superoxide dismutase; TGF, Transforming growth factor;↑, Increase or increase, ↓, Down or down; →, Stable or no change.

### 3.8. Arrhythmia

Arrhythmias are a common disease with complex etiology in clinical practice. The inducing factors include changes in myocardial tissue, conduction bundle, intense exercise, drug stimulation, electrolyte disorders, and so on (139). Arrhythmias have seriously affected the quality of life of the people. Currently, the main methods for treating arrhythmia include radiofrequency ablation, artificial pacemakers, and drug therapy (140). However, the current treatment of arrhythmia drugs and therapeutic efficacy are very limited. As one of the drugs that can effectively prevent and treat CVD, quercetin also has an important therapeutic effect on arrhythmia.

Quercetin (25 mg/kg) can be passed through TGF-β/Smads pathway inhibits myocardial fibrosis, thereby achieving the effect of treating arrhythmia (135). Quercetin can inhibit myocardial fibrosis and improve atrial fibrillation by regulating the expression of miR-223-3p/ Fork head Box Protein O3 (FOXO3) and activating autophagy (136) (Table 6). Pretreatment with quercetin 2 min before reperfusion arrhythmia can inhibit platelet aggregation and thromboxane A2 (TXA2) formation, thereby achieving the effect of preventing arrhythmia (141). As a commonly used antineoplastic drug, doxorubicin mainly increases cardiac toxicity by increasing LDH, iNOS, and NO. In the doxorubicin induced myocardial injury model, quercetin can significantly reduce the incidence of arrhythmia by increasing SOD activity, inhibiting iNOS and myocardial cell apoptosis (142). In addition, in the rat cardiomyopathy model, it was found that adding quercetin to drinking water can prevent the occurrence of lipid peroxidation in serum, thereby reducing arrhythmia (143). It can be seen that quercetin has sufficient experimental evidence in the treatment and prevention of arrhythmia, and the road from laboratory to clinical is not far away.

### 3.9. Antiplatelet

Antiplatelet therapy, an essential tool in the arsenal against myocardial infarction (MI) or heart attack, remains a critical component of modern cardiovascular medicine (144). Antiplatelet agents act as vital prophylactic and therapeutic measures by preventing the aggregation of platelets, crucial elements in clot formation and arterial blockage (145). Antiplatelet therapy encompasses a range of medications, such as aspirin, P2Y12 inhibitors and glycoprotein IIb/IIIa (GPIIb/IIIa) inhibitors, which have demonstrated their effectiveness in reducing MI and thrombotic complications risk (146). Furthermore, the potential of traditional Chinese medicine as an adjuvant treatment for MI is an emerging area of interest, with numerous studies investigating the therapeutic effects of various compounds and herbal formulations (147). By combining conventional antiplatelet therapies with alternative treatments, to ultimately reduce the global burden of MI and improve patient outcomes.

In vitro studies have shown that quercetin can inhibit platelet aggregation by several mechanisms. Firstly, it can effectively inhibit platelet activators adenosine diphosphate (ADP) and TXA2, thereby reducing the release of platelet particles (148). Second, it can inhibit the activation of platelet integrins, such as GPIIb/IIIa, which are essential for platelet aggregation (149). Third, it can interfere with the signaling pathways involved in platelet activation, such as the PI3K/Akt and MAPK pathways (150, 151). In addition, quercetin can also reduce platelet aggregation and thrombosis by inhibiting the PI3K/Akt and MAPK pathways (152).

Zaragozá et al. (153) found that quercetin has significant antiplatelet effects and a higher degree of COX enzyme inhibition. Perez et al. (154)investigated quercetin's vasodilatory, antiplatelet, and antiproliferative effects in hypertensive models. In a double-blind trial with 15 healthy volunteers, oral quercetin administration led to dose-dependent increases in quercetin-3-O-glucuronide (Q3GA) levels. No blood pressure changes were observed, but quercetin-induced brachial artery diameter increases were found to correlate with Q3GA levels and plasma glucuronidase activity. The study highlights quercetin's acute vasodilatory effects in individuals with normal blood pressure and cholesterol levels, consistent with Q3GA metabolite deconjugation.

In conclusion, quercetin has been shown to have antiplatelet effects *in vitro* and *in vivo*, by inhibiting platelet aggregation and thrombus formation through various mechanisms, including the inhibition of platelet granule release, integrin activation, and signaling pathways involved in platelet activation. These effects suggest that quercetin may have potential as a natural supplement to complement antiplatelet therapy and reduce the risk of adverse side effects associated with these medications. However, more clinical trials are needed to confirm these findings in humans and to determine the optimal dose and duration of quercetin supplementation. Further research is also needed to investigate the potential interactions between quercetin and antiplatelet medications, as well as the long-term effects of quercetin supplementation on cardiovascular outcomes.

### 3.10. CHD

CHD result from the narrowing or blockage of the coronary arteries responsible for supplying oxygen and nutrients to the heart muscle. This narrowing or blockage is primarily caused by the accumulation of plaque, consisting of cholesterol, fatty substances, and cellular waste products, within the arterial walls (155). CHD can lead to various complications such as complication is angina, characterized by chest pain or discomfort due to inadequate blood flow to the heart muscle. In more severe cases, CHD can result in myocardial infarction, heart failure, or even sudden cardiac death (156). The pathophysiology of CHD involves a complex interplay of processes, such as endothelial dysfunction, inflammation, and oxidative stress (157). Treatment for CHD typically involves a combination of lifestyle modifications, pharmacological interventions, and, in some cases, surgical procedures. Additionally, the role of specific micronutrients and functional foods, such as omega-3 fatty acids, antioxidants, and plant-based compounds, is being investigated for their potential cardioprotective properties (158–160).

Quercetin can inhibit the formation of CHD by attenuating oxidative stress and reducing the expression of adhesion molecules. It also can promote the vitality, migration, and angiogenesis of human microvascular endothelial cells by downregulating the expression of intercellular cell adhesion molecule-1 and Vascular cell adhesion molecule-1, and inhibit cell apoptosis (161). Abnormal lipid metabolism is one of the important risk factors for coronary heart disease. Quercetin can also regulate lipid metabolism by regulating the expression of key enzymes involved in cholesterol synthesis, such as 3-hydroxy-3-methyl glutaryl coenzyme A reductase (HMG-CoA) reductase, and is a new candidate drug for future development of cholesterol lowering drugs (137).

Although some studies have shown that quercetin has no impact on cardiovascular or thrombotic risk factors in healthy patients (162). However, other studies have found that treatment with 120 mg/day of quercetin can improve the ejection fraction of 88 CHD patients and reduce the frequency of ST segment changes and ventricular premature beats (106). However, more research is needed to confirm the optimal dosage and duration of quercetin. Furthermore, it is currently unclear whether there is a potential interaction between quercetin and existing cardiovascular drugs (Table 6).

### 3.11. Hyperlipemia

Persistent hyperlipidemia can cause the recruitment of inflammatory cells and the production of ROS by damaging the vascular endothelial function, thus leading to a series of cardiovascular and cerebrovascular events such as atherosclerosis, arterial stenosis, thrombosis and stroke (157, 163–165). Although lipid-lowering therapy is the main treatment for hyperlipidemia, these drugs can also cause side effects and are not sufficient to completely lower blood lipids (21, 166). Therefore, people are increasingly interested in alternative and complementary therapies for hyperlipidemia, including the use of traditional Chinese medicine and functional foods (167).

Zhang et al. (138) randomly divided 20 male Wistar rats into a control group and a quercetin supplementation group, and found that quercetin supplementation significantly increased the activity of hepatic cholesterol 7α-hydroxylase and the expression of ATP binding cassette transporter G1 mRNA and protein in the liver. Furthermore, it has been proven that quercetin can promote cholesterol efflux and promote the conversion of cholesterol into bile acids, thereby regulating liver cholesterol metabolism through these pathways.

Quercetin can also reduce the oxidation of low-density lipoprotein, thereby reducing the risk of developing hyperlipidemia (168). Janisch et al. (169) found that LDL oxidation lag time was increased by up to four times by low (<2 μM) concentrations of quercetin-3-glucuronide. Gnoni et al. (170) found that the formation of palmitic acid in rat hepatocytes treated with quercetin was significantly reduced after 30 min, indicating that quercetin has an inhibitory effect on fatty acid synthesis. The decrease in *de novo* synthesis of fatty acids and triacylglycerol (TAG) induced by quercetin, subsequently leading to a reduction in the formation of VLDL, may represent a potential mechanism underlying quercetin's ability to lower triacylglycerol levels.

Despite promising results *in vitro* and *in vivo* studies, there are relatively few clinical trials of quercetin for the prevention or treatment of human hyperlipidemia. A meta-analysis of five randomized controlled trials showed that quercetin did not significantly affect plasma LDL-C, HDL-C, and triglycerides (171). During subgroup analysis, it was also found that only plasma triglyceride levels were significantly correlated with the dosage and supplementation time of quercetin. Overall, the impact of quercetin on blood lipid levels is still uncertain, and its lipid-lowering effect may depend on the dosage and duration of supplementation.

## 4. Conclusion and outlook

Flavonoids are the main bioactive components of quercetin, which have multiple functions such as antioxidant, anti-inflammatory, myocardial protection, lipid lowering, blood pressure lowering, and improvement of myocardial ischemia and arrhythmia. However, the current research results on the mechanism and target of quercetin in the treatment of CVD are not uniform. The complex pharmacological actions and targets limit the application of quercetin in clinical patients. In addition, quercetin has the disadvantages of poor water solubility and low bioavailability. In order to further increase the pharmacological effects of quercetin, many structural modifications have been made to quercetin, mainly including the modification of hydroxyl groups to generate ethers and esters, the modification of carbonyl groups to generate carbonyl oxygen substituted products, and the modification of quercetin A and B rings. Quercetin derivatives with good solubility, high bioavailability, and significant biological activity were obtained through optimized modification (24, 172). Preparation of new dosage forms can also increase the pharmacological effects of quercetin, such as micro lotion, liposome encapsulation and nanocrystals.

In addition, the potential toxic side effects of quercetin may also be one of the reasons limiting its clinical application. However, in fact, among numerous published human intervention studies, the adverse reactions after supplementation with quercetin have been rarely reported, and even the reported adverse reactions are very mild (3). Although there are very few studies showing that prolonged and high-dose supplementation of quercetin can increase the risk of nephrotoxicity, it has not been found in human intervention experiments that quercetin increases nephrotoxicity in subjects with metabolic syndrome characteristics (19). Overall, oral administration of quercetin in humans appears to be well tolerated, with only a very low incidence of adverse reactions observed so far. However, this does not necessarily mean that quercetin has no toxic side effects, and more research is needed to confirm this.

Although researchers have made significant contributions to improving the bioavailability of quercetin. Many *in vitro* and *in vivo* studies have shown that quercetin has the effect of treating and preventing CVD, but there are still few clinical trials of quercetin in CVD, especially heart failure, myocardial infarction, ischemia reperfusion, myocardial hypertrophy, myocarditis, and other diseases. Even the doses and research results of quercetin used in clinical patients are uneven. In addition, it is not entirely clear which components of quercetin have practical applications, so it is necessary to further explore the monomer of traditional Chinese medicine. It can be seen that quercetin still needs a long way to be truly used in the treatment of patients with CVD. Firstly, it is necessary to focus on how to further improve the water solubility and oral bioavailability of quercetin. Secondly, the efficacy, mechanism of action, and unified application standards of quercetin in combination with other drugs. Finally, multicenter, large sample randomized controlled clinical trials are needed to further evaluate the safety and effectiveness of quercetin in CVD.

## References

[1] Roth GA Dwyer-Lindgren L Bertozzi-Villa A Stubbs RW Morozoff C Naghavi M Trends and patterns of geographic variation in cardiovascular mortality among US counties, 1980–2014. JAMA. (2017) 317(19):1976–92. 10.1001/jama.2017.4150 28510678 PMC5598768

[2] Ren J Fu L Nile SH Zhang J Kai G. Salvia miltiorrhiza in treating cardiovascular diseases: a review on its pharmacological and clinical applications. Front Pharmacol. (2019) 10:753. 10.3389/fphar.2019.00753 31338034 PMC6626924

[3] Andres S Pevny S Ziegenhagen R Bakhiya N Schäfer B Hirsch-Ernst KI Safety aspects of the use of quercetin as a dietary supplement. Mol Nutr Food Res. (2018) 62(1):10.1002/mnfr.201700447. 10.1002/mnfr.201700447 29127724

[4] Biler M Biedermann D Valentová K Křen V Kubala M. Quercetin and its analogues: optical and acido-basic properties. Phys Chem Chem Phys. (2017) 19(39):26870–9. 10.1039/C7CP03845C 28952614

[5] Hosseini A Razavi BM Banach M Hosseinzadeh H. Quercetin and metabolic syndrome: a review. Phytother Res. (2021) 35(10):5352–64. 10.1002/ptr.7144 34101925

[6] Patel RV Mistry BM Shinde SK Syed R Singh V Shin HS. Therapeutic potential of quercetin as a cardiovascular agent. Eur J Med Chem. (2018) 155:889–904. 10.1016/j.ejmech.2018.06.053 29966915

[7] Reyes-Farias M Carrasco-Pozo C. The anti-cancer effect of quercetin: molecular implications in cancer metabolism. Int J Mol Sci. (2019) 20(13):3177. 10.3390/ijms20133177 31261749 PMC6651418

[8] Shen P Lin W Deng X Ba X Han L Chen Z Potential implications of quercetin in autoimmune diseases. Front Immunol. (2021) 12:689044. 10.3389/fimmu.2021.689044 34248976 PMC8260830

[9] Geng L Liu Z Wang S Sun S Ma S Liu X Low-dose quercetin positively regulates mouse healthspan. Protein Cell. (2019) 10(10):770–5. 10.1007/s13238-019-0646-8 31325157 PMC6776572

[10] Zeng H Guo X Zhou F Xiao L Liu J Jiang C Quercetin alleviates ethanol-induced liver steatosis associated with improvement of lipophagy. Food Chem Toxicol. (2019) 125:21–8. 10.1016/j.fct.2018.12.028 30580029

[11] Li Y Cao R Mao Y Shao X Feng Y Zhai G. Research progress on structural modification and biological activity of quercetin. Chinese Tradit Herb Drugs. (2023) 54(5):1636–53. 10.7501/j.issn.0253-2670.2023.05.030

[12] Dehghani F Sezavar Seyedi Jandaghi SH Janani L Sarebanhassanabadi M Emamat H Vafa M. Effects of quercetin supplementation on inflammatory factors and quality of life in post-myocardial infarction patients: a double blind, placebo-controlled, randomized clinical trial. Phytother Res. (2021) 35(4):2085–98. 10.1002/ptr.6955 33216421

[13] Montenegro MF Neto-Neves EM Dias-Junior CA Ceron CS Castro MM Gomes VA Quercetin restores plasma nitrite and nitroso species levels in renovascular hypertension. Naunyn Schmiedebergs Arch Pharmacol. (2010) 382(4):293–301. 10.1007/s00210-010-0546-1 20694791

[14] Sánchez M Galisteo M Vera R Villar IC Zarzuelo A Tamargo J Quercetin downregulates NADPH oxidase, increases eNOS activity and prevents endothelial dysfunction in spontaneously hypertensive rats. J Hypertens. (2006) 24(1):75–84. 10.1097/01.hjh.0000198029.22472.d9 16331104

[15] Olaleye MT Crown OO Akinmoladun AC Akindahunsi AA. Rutin and quercetin show greater efficacy than nifedipin in ameliorating hemodynamic, redox, and metabolite imbalances in sodium chloride-induced hypertensive rats. Hum Exp Toxicol. (2014) 33(6):602–8. 10.1177/0960327113504790 24064906

[16] Prokosa MI. Indicators of endothelial dysfunction, markers of inflammation and lipid metabolism in patients with hypertension with the administration of quercetin. Wiad Lek. (2022) 75(7):1653–7. 10.36740/WLek202207107 35962675

[17] Kondratiuk VE Synytsia YP. Effect of quercetin on the echocardiographic parameters of left ventricular diastolic function in patients with gout and essential hypertension. Wiad Lek. (2018) 71(8):1554–9. 30684340

[18] Brüll V Burak C Stoffel-Wagner B Wolffram S Nickenig G Müller C Acute intake of quercetin from onion skin extract does not influence postprandial blood pressure and endothelial function in overweight-to-obese adults with hypertension: a randomized, double-blind, placebo-controlled, crossover trial. Eur J Nutr. (2017) 56(3):1347–57. 10.1007/s00394-016-1185-1 26924303

[19] Egert S Bosy-Westphal A Seiberl J Kürbitz C Settler U Plachta-Danielzik S Quercetin reduces systolic blood pressure and plasma oxidised low-density lipoprotein concentrations in overweight subjects with a high-cardiovascular disease risk phenotype: a double-blinded, placebo-controlled cross-over study. Br J Nutr. (2009) 102(7):1065–74. 10.1017/S0007114509359127 19402938

[20] Zahedi M Ghiasvand R Feizi A Asgari G Darvish L. Does quercetin improve cardiovascular risk factors and inflammatory biomarkers in women with type 2 diabetes: a double-blind randomized controlled clinical trial. Int J Prev Med. (2013) 4(7):777–85. 24049596 PMC3775217

[21] Pfeuffer M Auinger A Bley U Kraus-Stojanowic I Laue C Winkler P Effect of quercetin on traits of the metabolic syndrome, endothelial function and inflammation in men with different APOE isoforms. Nutr Metab Cardiovasc Dis. (2013) 23(5):403–9. 10.1016/j.numecd.2011.08.010 22118955

[22] Edwards RL Lyon T Litwin SE Rabovsky A Symons JD Jalili T. Quercetin reduces blood pressure in hypertensive subjects. J Nutr. (2007) 137(11):2405–11. 10.1093/jn/137.11.2405 17951477

[23] Ferenczyova K Kalocayova B Kindernay L Jelemensky M Balis P Berenyiova A Quercetin exerts age-dependent beneficial effects on blood pressure and vascular function, but is inefficient in preventing myocardial ischemia-reperfusion injury in zucker diabetic fatty rats. Molecules. (2020) 25(1):187. 10.3390/molecules25010187 31906454 PMC6983107

[24] Sharma A Kashyap D Sak K Tuli HS Sharma AK. Therapeutic charm of quercetin and its derivatives: a review of research and patents. Pharm Pat Anal. (2018) 7(1):15–32. 10.4155/ppa-2017-0030 29227203

[25] Ferenczyova K Kalocayova B Bartekova M. Potential implications of quercetin and its derivatives in cardioprotection. Int J Mol Sci. (2020) 21(5):1585. 10.3390/ijms21051585 PMC7084176 32111033

[26] Magar RT Sohng JK. A review on structure, modifications and structure-activity relation of quercetin and its derivatives. J Microbiol Biotechnol. (2020) 30(1):11–20. 10.4014/jmb.1907.07003 31752056 PMC9728256

[27] Jones RS Parker MD Morris ME. Quercetin, morin, luteolin, and phloretin are dietary flavonoid inhibitors of monocarboxylate transporter 6. Mol Pharm. (2017) 14(9):2930–6. 10.1021/acs.molpharmaceut.7b00264 28513167 PMC5585036

[28] Tang SM Deng XT Zhou J Li QP Ge XX Miao L. Pharmacological basis and new insights of quercetin action in respect to its anti-cancer effects. Biomed Pharmacother. (2020) 121:109604. 10.1016/j.biopha.2019.109604 31733570

[29] Limanaqi F Busceti CL Biagioni F Lazzeri G Forte M Schiavon S Cell clearing systems as targets of polyphenols in viral infections: potential implications for COVID-19 pathogenesis. Antioxidants (Basel). (2020) 9(11):1105. 10.3390/antiox9111105. PMC7697279 33182802

[30] D'Andrea G. Quercetin: a flavonol with multifaceted therapeutic applications? Fitoterapia. (2015) 106:256–71. 10.1016/j.fitote.2015.09.018 26393898

[31] Khan F Niaz K Maqbool F Ismail Hassan F Abdollahi M Nagulapalli Venkata KC Molecular targets underlying the anticancer effects of quercetin: an update. Nutrients. (2016) 8(9):529. 10.3390/nu8090529 27589790 PMC5037516

[32] Lewandowska H Kalinowska M Lewandowski W Stępkowski TM Brzóska K. The role of natural polyphenols in cell signaling and cytoprotection against cancer development. J Nutr Biochem. (2016) 32:1–19. 10.1016/j.jnutbio.2015.11.006 27142731

[33] Nijveldt RJ van Nood E van Hoorn DE Boelens PG van Norren K van Leeuwen PA. Flavonoids: a review of probable mechanisms of action and potential applications. Am J Clin Nutr. (2001) 74(4):418–25. 10.1093/ajcn/74.4.418 11566638

[34] Manach C Morand C Texier O Favier ML Agullo G Demigné C Quercetin metabolites in plasma of rats fed diets containing rutin or quercetin. J Nutr. (1995) 125(7):1911–22. 10.1093/jn/125.7.1911 7616308

[35] Russo M Spagnuolo C Tedesco I Bilotto S Russo GL. The flavonoid quercetin in disease prevention and therapy: facts and fancies. Biochem Pharmacol. (2012) 83(1):6–15. 10.1016/j.bcp.2011.08.010 21856292

[36] van der Woude H Boersma MG Vervoort J Rietjens IM. Identification of 14 quercetin phase II mono- and mixed conjugates and their formation by rat and human phase II in vitro model systems. Chem Res Toxicol. (2004) 17(11):1520–30. 10.1021/tx049826v 15540950

[37] Ulusoy HG Sanlier N. A minireview of quercetin: from its metabolism to possible mechanisms of its biological activities. Crit Rev Food Sci Nutr. (2020) 60(19):3290–303. 10.1080/10408398.2019.1683810 31680558

[38] Chen X Yin OQ Zuo Z Chow MS. Pharmacokinetics and modeling of quercetin and metabolites. Pharm Res. (2005) 22(6):892–901. 10.1007/s11095-005-4584-1 15948033

[39] Guo Y Bruno RS. Endogenous and exogenous mediators of quercetin bioavailability. J Nutr Biochem. (2015) 26(3):201–10. 10.1016/j.jnutbio.2014.10.008 25468612

[40] Arts IC Sesink AL Faassen-Peters M Hollman PC. The type of sugar moiety is a major determinant of the small intestinal uptake and subsequent biliary excretion of dietary quercetin glycosides. Br J Nutr. (2004) 91(6):841–7. 10.1079/BJN20041123 15182387

[41] Miltonprabu S Tomczyk M Skalicka-Woźniak K Rastrelli L Daglia M Nabavi SF Hepatoprotective effect of quercetin: from chemistry to medicine. Food Chem Toxicol. (2017) 108(Pt B):365–74. 10.1016/j.fct.2016.08.034 27591927

[42] Zaplatic E Bule M Shah SZA Uddin MS Niaz K. Molecular mechanisms underlying protective role of quercetin in attenuating Alzheimer’s disease. Life Sci. (2019) 224:109–19. 10.1016/j.lfs.2019.03.055 30914316

[43] Cione E La Torre C Cannataro R Caroleo MC Plastina P Gallelli L. Quercetin, epigallocatechin gallate, curcumin, and resveratrol: from dietary sources to human MicroRNA modulation. Molecules. (2019) 25(1):63. 10.3390/molecules25010063 31878082 PMC6983040

[44] Boots AW Haenen GR Bast A. Health effects of quercetin: from antioxidant to nutraceutical. Eur J Pharmacol. (2008) 585(2–3):325–37. 10.1016/j.ejphar.2008.03.008 18417116

[45] Min Z Yangchun L Yuquan W Changying Z. Quercetin inhibition of myocardial fibrosis through regulating MAPK signaling pathway via ROS. Pak J Pharm Sci. (2019) 32(3 Special):1355–9. 31551215

[46] Lu XL Zhao CH Yao XL Zhang H. Quercetin attenuates high fructose feeding-induced atherosclerosis by suppressing inflammation and apoptosis via ROS-regulated PI3K/AKT signaling pathway. Biomed Pharmacother. (2017) 85:658–71. 10.1016/j.biopha.2016.11.077 27919735

[47] Saw CL Guo Y Yang AY Paredes-Gonzalez X Ramirez C Pung D The berry constituents quercetin, kaempferol, and pterostilbene synergistically attenuate reactive oxygen species: involvement of the Nrf2-ARE signaling pathway. Food Chem Toxicol. (2014) 72:303–11. 10.1016/j.fct.2014.07.038 25111660

[48] d’ Avila Farias M Oliveira PS Dutra FS Fernandes TJ de Pereira CM de Oliveira SQ Eugenol derivatives as potential anti-oxidants: is phenolic hydroxyl necessary to obtain an effect? J Pharm Pharmacol. (2014) 66(5):733–46. 10.1111/jphp.12197 24372555

[49] Li B Yang M Liu JW Yin GT. Protective mechanism of quercetin on acute myocardial infarction in rats. Genet Mol Res. (2016) 15(1):15017117. 10.4238/gmr.15017117 26985950

[50] Milton Prabu S Muthumani M Shagirtha K. Quercetin potentially attenuates cadmium induced oxidative stress mediated cardiotoxicity and dyslipidemia in rats. Eur Rev Med Pharmacol Sci. (2013) 17(5):582–95. 23543441

[51] Valko M Jomova K Rhodes CJ Kuča K Musílek K. Redox- and non-redox-metal-induced formation of free radicals and their role in human disease. Arch Toxicol. (2016) 90(1):1–37. 10.1007/s00204-015-1579-5 26343967

[52] Cherrak SA Mokhtari-Soulimane N Berroukeche F Bensenane B Cherbonnel A Merzouk H In vitro antioxidant versus metal Ion chelating properties of flavonoids: a structure-activity investigation. PLoS One. (2016) 11(10):e0165575. 10.1371/journal.pone.0165575 27788249 PMC5082868

[53] Babenkova IV Osipov AN Teselkin YO. The effect of dihydroquercetin on catalytic activity of iron (II) ions in the fenton reaction. Bull Exp Biol Med. (2018) 165(3):347–50. 10.1007/s10517-018-4167-x 30006874

[54] Tang Y Li Y Yu H Gao C Liu L Xing M Quercetin attenuates chronic ethanol hepatotoxicity: implication of “free” iron uptake and release. Food Chem Toxicol. (2014) 67:131–8. 10.1016/j.fct.2014.02.022 24569067

[55] Pękal A Biesaga M Pyrzynska K. Interaction of quercetin with copper ions: complexation, oxidation and reactivity towards radicals. Biometals. (2011) 24(1):41–9. 10.1007/s10534-010-9372-7 20835752

[56] Jomova K Lawson M Drostinova L Lauro P Poprac P Brezova V Protective role of quercetin against copper(II)-induced oxidative stress: a spectroscopic, theoretical and DNA damage study. Food Chem Toxicol. (2017) 110:340–50. 10.1016/j.fct.2017.10.042 29107026

[57] Kattoor AJ Kanuri SH Mehta JL. Role of ox-LDL and LOX-1 in atherogenesis. Curr Med Chem. (2019) 26(9):1693–700. 10.2174/0929867325666180508100950 29737246

[58] Pirillo A Norata GD Catapano AL. LOX-1, OxLDL, and atherosclerosis. Mediators Inflamm. (2013) 2013:152786. 10.1155/2013/152786 23935243 PMC3723318

[59] Chistiakov DA Kashirskikh DA Khotina VA Grechko AV Orekhov AN. Immune-Inflammatory responses in atherosclerosis: the role of myeloid cells. J Clin Med. (2019) 8(11):1798. 10.3390/jcm8111798 31717832 PMC6912749

[60] Jiang YH Jiang LY Wang YC Ma DF Li X. Quercetin attenuates atherosclerosis via modulating oxidized LDL-induced endothelial cellular senescence. Front Pharmacol. (2020) 11:512. 10.3389/fphar.2020.00512 32410992 PMC7198817

[61] Hertog MG Feskens EJ Hollman PC Katan MB Kromhout D. Dietary flavonoids and cancer risk in the zutphen elderly study. Nutr Cancer. (1994) 22(2):175–84. 10.1080/01635589409514342 14502846

[62] Liang Q Chen Y Li C Lu L. Quercetin attenuates ox-LDL-induced calcification in vascular smooth muscle cells by regulating ROS-TLR4 signaling pathway. Nan Fang Yi Ke Da Xue Xue Bao. (2018) 38(8):980–5. 10.3969/j.issn.1673-4254.2018.08.13 30187880 PMC6744032

[63] Pedro-Botet J Climent E Benaiges D. Atherosclerosis and inflammation. New therapeutic approaches. Med Clin (Barc). (2020) 155(6):256–62. 10.1016/j.medcli.2020.04.024 32571617

[64] Pang J Hu P Wang J Jiang J Lai J. Vorapaxar stabilizes permeability of the endothelial barrier under cholesterol stimulation via the AKT/JNK and NF-κB signaling pathways. Mol Med Rep. (2019) 19(6):5291–300. 31059055 10.3892/mmr.2019.10211 PMC6522885

[65] Lee JY Park W. Anti-Inflammatory effect of wogonin on RAW 264.7 mouse macrophages induced with polyinosinic-polycytidylic acid. Molecules. (2015) 20(4):6888–900. 10.3390/molecules20046888 25913928 PMC6273246

[66] Kondo M Izawa-Ishizawa Y Goda M Hosooka M Kagimoto Y Saito N Preventive effects of quercetin against the onset of atherosclerosis-related acute aortic syndromes in mice. Int J Mol Sci. (2020) 21(19):7226. 10.3390/ijms21197226 PMC7582618 33007902

[67] Zhang LL Zhang HT Cai YQ Han YJ Yao F Yuan ZH Anti-inflammatory effect of mesenchymal stromal cell transplantation and quercetin treatment in a rat model of experimental cerebral ischemia. Cell Mol Neurobiol. (2016) 36(7):1023–34. 10.1007/s10571-015-0291-6 27008429 PMC11482404

[68] Si TL Liu Q Ren YF Li H Xu XY Li EH Enhanced anti-inflammatory effects of DHA and quercetin in lipopolysaccharide-induced RAW264.7 macrophages by inhibiting NF-κB and MAPK activation. Mol Med Rep. (2016) 14(1):499–508. 10.3892/mmr.2016.5259 27176922

[69] Song L Xu M Lopes-Virella MF Huang Y. Quercetin inhibits matrix metalloproteinase-1 expression in human vascular endothelial cells through extracellular signal-regulated kinase. Arch Biochem Biophys. (2001) 391(1):72–8. 10.1006/abbi.2001.2402 11414687

[70] Saragusti AC Ortega MG Cabrera JL Estrin DA Marti MA Chiabrando GA. Inhibitory effect of quercetin on matrix metalloproteinase 9 activity molecular mechanism and structure-activity relationship of the flavonoid-enzyme interaction. Eur J Pharmacol. (2010) 644(1-3):138–45. 10.1016/j.ejphar.2010.07.001 20619256

[71] Xiao L Liu L Guo X Zhang S Wang J Zhou F Quercetin attenuates high fat diet-induced atherosclerosis in apolipoprotein E knockout mice: a critical role of NADPH oxidase. Food Chem Toxicol. (2017) 105:22–33. 10.1016/j.fct.2017.03.048 28351769

[72] Shen Y Ward NC Hodgson JM Puddey IB Wang Y Zhang D Dietary quercetin attenuates oxidant-induced endothelial dysfunction and atherosclerosis in apolipoprotein E knockout mice fed a high-fat diet: a critical role for heme oxygenase-1. Free Radic Biol Med. (2013) 65:908–15. 10.1016/j.freeradbiomed.2013.08.185 24017971

[73] Garelnabi M Mahini H Wilson T. Quercetin intake with exercise modulates lipoprotein metabolism and reduces atherosclerosis plaque formation. J Int Soc Sports Nutr. (2014) 11:22. 10.1186/1550-2783-11-22 24890098 PMC4041042

[74] Padma VV Lalitha G Shirony NP Baskaran R. Effect of quercetin against lindane induced alterations in the serum and hepatic tissue lipids in wistar rats. Asian Pac J Trop Biomed. (2012) 2(11):910–5. 10.1016/S2221-1691(12)60252-4 23569870 PMC3609243

[75] Thygesen K Alpert JS Jaffe AS Chaitman BR Bax JJ Morrow DA Fourth universal definition of myocardial infarction. J Am Coll Cardiol. (2018) 72(18):2231–64. 10.1016/j.jacc.2018.08.1038 30153967

[76] Fedorchenko MV Seredyuk NM Petrovskyi RV. Influence of trimetazidine and levocarnitine on clinical course, structural and functional changes and myocardial fibrosis in patients with myocardial infarction. Wiad Lek. (2019) 72(11 cz 1):2094–8. 31860853

[77] Stewart RAH Held C Hadziosmanovic N Armstrong PW Cannon CP Granger CB Physical activity and mortality in patients with stable coronary heart disease. J Am Coll Cardiol. (2017) 70(14):1689–700. 10.1016/j.jacc.2017.08.017 28958324

[78] Teekakirikul P Zhu W Huang HC Fung E. Hypertrophic cardiomyopathy: an overview of genetics and management. Biomolecules. (2019) 9(12):878. 10.3390/biom9120878 31888115 PMC6995589

[79] Tomé-Carneiro J Visioli F. Polyphenol-based nutraceuticals for the prevention and treatment of cardiovascular disease: review of human evidence. Phytomedicine. (2016) 23(11):1145–74. 10.1016/j.phymed.2015.10.018 26776959

[80] Lin QY Lang PP Zhang YL Yang XL Xia YL Bai J Pharmacological blockage of ICAM-1 improves angiotensin II-induced cardiac remodeling by inhibiting adhesion of LFA-1(+) monocytes. Am J Physiol Heart Circ Physiol. (2019) 317(6):H1301–h11. 10.1152/ajpheart.00566.2019 31729904

[81] Wang L Tan A An X Xia Y Xie Y. Quercetin dihydrate inhibition of cardiac fibrosis induced by angiotensin II in vivo and in vitro. Biomed Pharmacother. (2020) 127:110205. 10.1016/j.biopha.2020.110205 32403046

[82] Hung CH Chan SH Chu PM Tsai KL. Quercetin is a potent anti-atherosclerotic compound by activation of SIRT1 signaling under oxLDL stimulation. Mol Nutr Food Res. (2015) 59(10):1905–17. 10.1002/mnfr.201500144 26202455

[83] Zhang F Feng J Zhang J Kang X Qian D. Quercetin modulates AMPK/SIRT1/NF-κB signaling to inhibit inflammatory/oxidative stress responses in diabetic high fat diet-induced atherosclerosis in the rat carotid artery. Exp Ther Med. (2020) 20(6):280. 10.3892/etm.2020.9410 33200005 PMC7664594

[84] de Lacerda Alexandre JV Viana YIP David CEB Cunha PLO Albuquerque AC Varela ALN Quercetin treatment increases H(2)O(2) removal by restoration of endogenous antioxidant activity and blocks isoproterenol-induced cardiac hypertrophy. Naunyn Schmiedebergs Arch Pharmacol. (2021) 394(2):217–26. 10.1007/s00210-020-01953-8 32930861

[85] Sharma A Parikh M Shah H Gandhi T. Modulation of Nrf2 by quercetin in doxorubicin-treated rats. Heliyon. (2020) 6(4):e03803. 10.1016/j.heliyon.2020.e03803 32337383 PMC7177035

[86] Albadrani GM BinMowyna MN Bin-Jumah MN El-Akabawy G Aldera H Al-Farga AM. Quercetin prevents myocardial infarction adverse remodeling in rats by attenuating TGF-β1/Smad3 signaling: different mechanisms of action. Saudi J Biol Sci. (2021) 28(5):2772–82. 10.1016/j.sjbs.2021.02.007 34012318 PMC8116976

[87] Albadrani GM Binmowyna MN Bin-Jumah MN El-Akabawy G Aldera H Al-Farga AM. Quercetin protects against experimentally-induced myocardial infarction in rats by an antioxidant potential and concomitant activation of signal transducer and activator of transcription 3. J Physiol Pharmacol. (2020) 71(6). 10.26402/jpp.2020.6.11 33901998

[88] Lu TM Chiu HF Shen YC Chung CC Venkatakrishnan K Wang CK. Hypocholesterolemic efficacy of quercetin rich onion juice in healthy mild hypercholesterolemic adults: a pilot study. Plant Foods Hum Nutr. (2015) 70(4):395–400. 10.1007/s11130-015-0507-4 26385226

[89] Javadi F Eghtesadi S Ahmadzadeh A Aryaeian N Zabihiyeganeh M Foroushani AR The effect of quercetin on plasma oxidative status, C-reactive protein and blood pressure in women with rheumatoid arthritis. Int J Prev Med. (2014) 5(3):293–301. 24829713 PMC4018638

[90] Heinz SA Henson DA Nieman DC Austin MD Jin F. A 12-week supplementation with quercetin does not affect natural killer cell activity, granulocyte oxidative burst activity or granulocyte phagocytosis in female human subjects. Br J Nutr. (2010) 104(6):849–57. 10.1017/S000711451000156X 20500927

[91] Lee KH Park E Lee HJ Kim MO Cha YJ Kim JM Effects of daily quercetin-rich supplementation on cardiometabolic risks in male smokers. Nutr Res Pract. (2011) 5(1):28–33. 10.4162/nrp.2011.5.1.28 21487493 PMC3061266

[92] Annapurna A Reddy CS Akondi RB Rao SR. Cardioprotective actions of two bioflavonoids, quercetin and rutin, in experimental myocardial infarction in both normal and streptozotocin-induced type I diabetic rats. J Pharm Pharmacol. (2009) 61(10):1365–74. 10.1211/jpp.61.10.0014 19814870

[93] Yu H Zhang H Zhao W Guo L Li X Li Y Gypenoside protects against myocardial ischemia-reperfusion injury by inhibiting cardiomyocytes apoptosis via inhibition of CHOP pathway and activation of PI3K/akt pathway in vivo and in vitro. Cell Physiol Biochem. (2016) 39(1):123–36. 10.1159/000445611 27322831

[94] Kong Q Dai L Wang Y Zhang X Li C Jiang S HSPA12B Attenuated acute myocardial ischemia/reperfusion injury via maintaining endothelial integrity in a PI3K/akt/mTOR-dependent mechanism. Sci Rep. (2016) 6:33636. 10.1038/srep33636 27644317 PMC5028890

[95] Sanhueza J Valdes J Campos R Garrido A Valenzuela A. Changes in the xanthine dehydrogenase/xanthine oxidase ratio in the rat kidney subjected to ischemia-reperfusion stress: preventive effect of some flavonoids. Res Commun Chem Pathol Pharmacol. (1992) 78(2):211–8. 1475527

[96] Chen YW Chou HC Lin ST Chen YH Chang YJ Chen L Cardioprotective effects of quercetin in cardiomyocyte under ischemia/reperfusion injury. Evid Based Complement Alternat Med. (2013) 2013:364519. 10.1155/2013/364519 23573126 PMC3612448

[97] Bartekova M Radosinska J Pancza D Barancik M Ravingerova T. Cardioprotective effects of quercetin against ischemia-reperfusion injury are age-dependent. Physiol Res. (2016) 65(Suppl 1):S101–7. 10.33549/physiolres.933390 27643931

[98] Dong LY Chen F Xu M Yao LP Zhang YJ Zhuang Y. Quercetin attenuates myocardial ischemia-reperfusion injury via downregulation of the HMGB1-TLR4-NF-κB signaling pathway. Am J Transl Res. (2018) 10(5):1273–83. 29887944 PMC5992549

[99] Liu Y Song Y Li S Mo L. Cardioprotective effect of quercetin against ischemia/reperfusion injury is mediated through NO system and mitochondrial K-ATP channels. Cell J. (2021) 23(2):184–90. 10.22074/cellj.2021.7183 34096219 PMC8181321

[100] Wang Y Zhang ZZ Wu Y Ke JJ He XH Wang YL. Quercetin postconditioning attenuates myocardial ischemia/reperfusion injury in rats through the PI3K/akt pathway. Braz J Med Biol Res. (2013) 46(10):861–7. 10.1590/1414-431X20133036 24068165 PMC3854307

[101] Liu H Guo X Chu Y Lu S. Heart protective effects and mechanism of quercetin preconditioning on anti-myocardial ischemia reperfusion (IR) injuries in rats. Gene. (2014) 545(1):149–55. 10.1016/j.gene.2014.04.043 24769323

[102] Jin HB Yang YB Song YL Zhang YC Li YR. Protective roles of quercetin in acute myocardial ischemia and reperfusion injury in rats. Mol Biol Rep. (2012) 39(12):11005–9. 10.1007/s11033-012-2002-4 23053990

[103] Ahmed LA Salem HA Attia AS El-Sayed ME. Enhancement of amlodipine cardioprotection by quercetin in ischaemia/reperfusion injury in rats. J Pharm Pharmacol. (2009) 61(9):1233–41. 10.1211/jpp.61.09.0014 19703374

[104] Wan LL Xia J Ye D Liu J Chen J Wang G. Effects of quercetin on gene and protein expression of NOX and NOS after myocardial ischemia and reperfusion in rabbit. Cardiovasc Ther. (2009) 27(1):28–33. 10.1111/j.1755-5922.2009.00071.x 19207477

[105] Brookes PS Digerness SB Parks DA Darley-Usmar V. Mitochondrial function in response to cardiac ischemia-reperfusion after oral treatment with quercetin. Free Radic Biol Med. (2002) 32(11):1220–8. 10.1016/S0891-5849(02)00839-0 12031906

[106] Chekalina NI Shut SV Trybrat TA Burmak YH Petrov YY Manusha YI Effect of quercetin on parameters of central hemodynamics and myocardial ischemia in patients with stable coronary heart disease. Wiad Lek. (2017) 70(4):707–11. 29064791

[107] Malishevskaia IV Ilashchuk TA Okipniak IV. Therapeutic efficacy of quercetin in patients with is ischemic heart disease with underlying metabolic syndrome. Georgian Med News. (2013) 225:67–71. 24423679

[108] Liu CJ Yao L Hu YM Zhao BT. Effect of quercetin-loaded mesoporous silica nanoparticles on myocardial ischemia-reperfusion injury in rats and its mechanism. Int J Nanomed. (2021) 16:741–52. 10.2147/IJN.S277377 PMC7866914 33564233

[109] Tang J Lu L Liu Y Ma J Yang L Li L Quercetin improve ischemia/reperfusion-induced cardiomyocyte apoptosis in vitro and in vivo study via SIRT1/PGC-1α signaling. J Cell Biochem. (2019) 120(6):9747–57. 10.1002/jcb.28255 30656723

[110] Yan L Zhang JD Wang B Lv YJ Jiang H Liu GL Quercetin inhibits left ventricular hypertrophy in spontaneously hypertensive rats and inhibits angiotensin II-induced H9C2 cells hypertrophy by enhancing PPAR-*γ* expression and suppressing AP-1 activity. PLoS One. (2013) 8(9):e72548. 10.1371/journal.pone.0072548 24039778 PMC3769399

[111] Ruwhof C van der Laarse A. Mechanical stress-induced cardiac hypertrophy: mechanisms and signal transduction pathways. Cardiovasc Res. (2000) 47(1):23–37. 10.1016/S0008-6363(00)00076-6 10869527

[112] Wang Y Wang HY Yuan ZK Zhao XN Wang JX Zhang ZX. Quercetin decreased heart rate and cardiomyocyte Ca2 + oscillation frequency in rats and prevented cardiac hypertrophy in mice. Zhongguo Yao Li Xue Bao. (1999) 20(5):426–30. 10678090

[113] Jalili T Carlstrom J Kim S Freeman D Jin H Wu TC Quercetin-supplemented diets lower blood pressure and attenuate cardiac hypertrophy in rats with aortic constriction. J Cardiovasc Pharmacol. (2006) 47(4):531–41. 10.1097/01.fjc.0000211746.78454.50 16680066

[114] Qin TC Chen L Yu LX Gu ZL. Inhibitory effect of quercetin on cultured neonatal rat cardiomyocytes hypertrophy induced by angiotensin. Acta Pharmacol Sin. (2001) 22(12):1103–6. 11749808

[115] Han JJ Hao J Kim CH Hong JS Ahn HY Lee YS. Quercetin prevents cardiac hypertrophy induced by pressure overload in rats. J Vet Med Sci. (2009) 71(6):737–43. 10.1292/jvms.71.737 19578281

[116] Ulasova E Perez J Hill BG Bradley WE Garber DW Landar A Quercetin prevents left ventricular hypertrophy in the apo E knockout mouse. Redox Biol. (2013) 1(1):381–6. 10.1016/j.redox.2013.07.001 24024175 PMC3757709

[117] Chen K Rekep M Wei W Wu Q Xue Q Li S Quercetin prevents in vivo and in vitro myocardial hypertrophy through the proteasome-GSK-3 pathway. Cardiovasc Drugs Ther. (2018) 32(1):5–21. 10.1007/s10557-018-6771-4 29435775

[118] Miao C Chang J Zhang G. Recent research progress of microRNAs in hypertension pathogenesis, with a focus on the roles of miRNAs in pulmonary arterial hypertension. Mol Biol Rep. (2018) 45(6):2883–96. 10.1007/s11033-018-4335-0 30298350

[119] Ramirez LA Sullivan JC. Sex differences in hypertension: where we have been and where we are going. Am J Hypertens. (2018) 31(12):1247–54. 10.1093/ajh/hpy148 30299518 PMC6233684

[120] Kim SG Kim JR Choi HC. Quercetin-Induced AMP-activated protein kinase activation attenuates vasoconstriction through LKB1-AMPK signaling pathway. J Med Food. (2018) 21(2):146–53. 10.1089/jmf.2017.4052 29035613

[121] Lin X Han T Fan Y Wu S Wang F Wang C. Quercetin improves vascular endothelial function through promotion of autophagy in hypertensive rats. Life Sci. (2020) 258:118106. 10.1016/j.lfs.2020.118106 32682916

[122] Pereira SC Parente JM Belo VA Mendes AS Gonzaga NA do Vale GT Quercetin decreases the activity of matrix metalloproteinase-2 and ameliorates vascular remodeling in renovascular hypertension. Atherosclerosis. (2018) 270:146–53. 10.1016/j.atherosclerosis.2018.01.031 29425960

[123] Mackraj I Govender T Ramesar S. The antihypertensive effects of quercetin in a salt-sensitive model of hypertension. J Cardiovasc Pharmacol. (2008) 51(3):239–45. 10.1097/FJC.0b013e318162011f 18356687

[124] Duarte J Galisteo M Ocete MA Pérez-Vizcaino F Zarzuelo A Tamargo J. Effects of chronic quercetin treatment on hepatic oxidative status of spontaneously hypertensive rats. Mol Cell Biochem. (2001) 221(1–2):155–60. 10.1023/A:1010956928584 11506179

[125] Duarte J Pérez-Palencia R Vargas F Ocete MA Pérez-Vizcaino F Zarzuelo A Antihypertensive effects of the flavonoid quercetin in spontaneously hypertensive rats. Br J Pharmacol. (2001) 133(1):117–24. 10.1038/sj.bjp.0704064 11325801 PMC1572775

[126] Sun X Zhang S Song H. Quercetin attenuates reduced uterine perfusion pressure -induced hypertension in pregnant rats through regulation of endothelin-1 and endothelin-1 type A receptor. Lipids Health Dis. (2020) 19(1):180. 10.1186/s12944-020-01357-w 32758232 PMC7409636

[127] Ajibade TO Oyagbemi AA Omobowale TO Asenuga ER Adigun KO. Quercetin and vitamin C mitigate cobalt chloride-induced hypertension through reduction in oxidative stress and nuclear factor kappa Beta (NF-kb) expression in experimental rat model. Biol Trace Elem Res. (2017) 175(2):347–59. 10.1007/s12011-016-0773-5 27283837

[128] Maksymchuk O Shysh A Kotliarova A. Quercetin inhibits the expression of MYC and CYP2E1 and reduces oxidative stress in the myocardium of spontaneously hypertensive rats. Acta Biochim Pol. (2023) 70(1):199–204. 10.18388/abp.2020_6517 36729410

[129] Oyagbemi AA Omobowale TO Ola-Davies OE Asenuga ER Ajibade TO Adejumobi OA Quercetin attenuates hypertension induced by sodium fluoride via reduction in oxidative stress and modulation of HSP 70/ERK/PPAR*γ* signaling pathways. Biofactors. (2018) 44(5):465–79. 10.1002/biof.1445 30171731

[130] Larson A Witman MA Guo Y Ives S Richardson RS Bruno RS Acute, quercetin-induced reductions in blood pressure in hypertensive individuals are not secondary to lower plasma angiotensin-converting enzyme activity or endothelin-1: nitric oxide. Nutr Res. (2012) 32(8):557–64. 10.1016/j.nutres.2012.06.018 22935338

[131] Tan X Xian W Chen Y Li X Wang Q Kang P Exploring the therapeutic mechanism of quercetin for heart failure based on network pharmacology and molecular docking. Nan Fang Yi Ke Da Xue Xue Bao. (2021) 41(8):1198–206. 10.12122/j.issn.1673-4254.2021.08.11 34549711 PMC8527225

[132] Wang SH Tsai KL Chou WC Cheng HC Huang YT Ou HC Quercetin mitigates cisplatin-induced oxidative damage and apoptosis in cardiomyocytes through Nrf2/HO-1 signaling pathway. Am J Chin Med. (2022) 50(5):1281–98. 10.1142/S0192415X22500537 35670059

[133] Falk E. Pathogenesis of atherosclerosis. J Am Coll Cardiol. (2006) 47(8 Suppl):C7–12. 10.1016/j.jacc.2005.09.068 16631513

[134] Chang X Zhang T Wang J Liu Y Yan P Meng Q SIRT5-Related Desuccinylation modification contributes to quercetin-induced protection against heart failure and high-glucose-prompted cardiomyocytes injured through regulation of mitochondrial quality surveillance. Oxid Med Cell Longev. (2021) 2021:5876841. 10.1155/2021/5876841 34603599 PMC8486530

[135] Wang H Jiang W Hu Y Wan Z Bai H Yang Q Quercetin improves atrial fibrillation through inhibiting TGF-β/smads pathway via promoting MiR-135b expression. Phytomedicine. (2021) 93:153774. 10.1016/j.phymed.2021.153774 34656066

[136] Hu J Wang X Cui X Kuang W Li D Wang J. Quercetin prevents isoprenaline-induced myocardial fibrosis by promoting autophagy via regulating miR-223-3p/FOXO3. Cell Cycle. (2021) 20(13):1253–69. 10.1080/15384101.2021.1932029 34097559 PMC8331011

[137] Khamis AA Salama AF Kenawy ME Mohamed TM. Regulation of hepatic hydroxy methyl glutarate—coA reductase for controlling hypercholesterolemia in rats. Biomed Pharmacother. (2017) 95:1242–50. 10.1016/j.biopha.2017.09.071 28938515

[138] Zhang M Xie Z Gao W Pu L Wei J Guo C. Quercetin regulates hepatic cholesterol metabolism by promoting cholesterol-to-bile acid conversion and cholesterol efflux in rats. Nutr Res. (2016) 36(3):271–9. 10.1016/j.nutres.2015.11.019 26923514

[139] Garcia-Elias A Benito B. Ion channel disorders and sudden cardiac death. Int J Mol Sci. (2018) 19(3):692. 10.3390/ijms19030692 29495624 PMC5877553

[140] Zhou Y Suo W Zhang X Lv J Liu Z Liu R. Roles and mechanisms of quercetin on cardiac arrhythmia: a review. Biomed Pharmacother. (2022) 153:113447. 10.1016/j.biopha.2022.113447 36076562

[141] Xiao D Gu ZL Qian ZN. Effects of quercetin on platelet and reperfusion-induced arrhythmias in rats. Zhongguo Yao Li Xue Bao. (1993) 14(6):505–8. 8010047

[142] Pei TX Xu CQ Li B Zhang ZR Gao XX Yu J Protective effect of quercetin against Adriamycin-induced cardiotoxicity and its mechanism in mice. Yao Xue Xue Bao. (2007) 42(10):1029–33. 18229606

[143] Khunderyakova NV Belosludtseva NV Khmil NV Mosentsov AA Stepanov MR Ananyan MA [Effect of per os administration of dihydroquercetin aqueous form on energy exchange in blood lymphocytes of rats with experimental cardiomyopathy]. Vopr Pitan. (2021) 90(6):50–8. 10.33029/0042-8833-2021-90-6-50-58 35032124

[144] Jackson SP Schoenwaelder SM. Antiplatelet therapy: in search of the ‘magic bullet’. Nat Rev Drug Discov. (2003) 2(10):775–89. 10.1038/nrd1198 14526381

[145] Davì G Patrono C. Platelet activation and atherothrombosis. N Engl J Med. (2007) 357(24):2482–94. 10.1056/NEJMra071014 18077812

[146] Wallentin L Becker RC Budaj A Cannon CP Emanuelsson H Held C Ticagrelor versus clopidogrel in patients with acute coronary syndromes. N Engl J Med. (2009) 361(11):1045–57. 10.1056/NEJMoa0904327 19717846

[147] Hao P Jiang F Cheng J Ma L Zhang Y Zhao Y. Traditional Chinese medicine for cardiovascular disease: evidence and potential mechanisms. J Am Coll Cardiol. (2017) 69(24):2952–66. 10.1016/j.jacc.2017.04.041 28619197

[148] Guerrero JA Lozano ML Castillo J Benavente-García O Vicente V Rivera J. Flavonoids inhibit platelet function through binding to the thromboxane A2 receptor. J Thromb Haemost. (2005) 3(2):369–76. 10.1111/j.1538-7836.2004.01099.x 15670046

[149] Zaragozá C Monserrat J Mantecón C Villaescusa L Álvarez-Mon M Zaragozá F Binding and antiplatelet activity of quercetin, rutin, diosmetin, and diosmin flavonoids. Biomed Pharmacother. (2021) 141:111867. 10.1016/j.biopha.2021.111867 34229245

[150] Endale M Park SC Kim S Kim SH Yang Y Cho JY Quercetin disrupts tyrosine-phosphorylated phosphatidylinositol 3-kinase and myeloid differentiation factor-88 association, and inhibits MAPK/AP-1 and IKK/NF-κB-induced inflammatory mediators production in RAW 264.7 cells. Immunobiology. (2013) 218(12):1452–67. 10.1016/j.imbio.2013.04.019 23735482

[151] Oh WJ Endale M Park SC Cho JY Rhee MH. Dual roles of quercetin in platelets: phosphoinositide-3-kinase and MAP kinases inhibition, and cAMP-dependent vasodilator-stimulated phosphoprotein stimulation. Evid Based Complement Alternat Med. (2012) 2012:485262. 10.1155/2012/485262 23304202 PMC3533481

[152] Navarro-Núñez L Lozano ML Martínez C Vicente V Rivera J. Effect of quercetin on platelet spreading on collagen and fibrinogen and on multiple platelet kinases. Fitoterapia. (2010) 81(2):75–80. 10.1016/j.fitote.2009.08.006 19686810

[153] Zaragozá C Álvarez-Mon M Zaragozá F Villaescusa L. Flavonoids: antiplatelet effect as inhibitors of COX-1. Molecules. (2022) 27(3):1146. 10.3390/molecules27031146 PMC8839657 35164411

[154] Perez A Gonzalez-Manzano S Jimenez R Perez-Abud R Haro JM Osuna A The flavonoid quercetin induces acute vasodilator effects in healthy volunteers: correlation with beta-glucuronidase activity. Pharmacol Res. (2014) 89:11–8. 10.1016/j.phrs.2014.07.005 25076013

[155] Benjamin EJ Muntner P Alonso A Bittencourt MS Callaway CW Carson AP Heart disease and stroke statistics-2019 update: a report from the American heart association. Circulation. (2019) 139(10):e56–e528. 10.1161/CIR.0000000000000659 30700139

[156] Roger VL Weston SA Gerber Y Killian JM Dunlay SM Jaffe AS Trends in incidence, severity, and outcome of hospitalized myocardial infarction. Circulation. (2010) 121(7):863–9. 10.1161/CIRCULATIONAHA.109.897249 20142444 PMC2827641

[157] Libby P Ridker PM Hansson GK. Progress and challenges in translating the biology of atherosclerosis. Nature. (2011) 473(7347):317–25. 10.1038/nature10146 21593864

[158] Manson JE Cook NR Lee IM Christen W Bassuk SS Mora S Marine n-3 fatty acids and prevention of cardiovascular disease and cancer. N Engl J Med. (2019) 380(1):23–32. 10.1056/NEJMoa1811403 30415637 PMC6392053

[159] Liguori I Russo G Curcio F Bulli G Aran L Della-Morte D Oxidative stress, aging, and diseases. Clin Interv Aging. (2018) 13:757–72. 10.2147/CIA.S158513 29731617 PMC5927356

[160] Tressera-Rimbau A Arranz S Eder M Vallverdú-Queralt A. Dietary polyphenols in the prevention of stroke. Oxid Med Cell Longev. (2017) 2017:7467962. 10.1155/2017/7467962 29204249 PMC5674514

[161] Li MT Ke J Guo SF Wu Y Bian YF Shan LL The protective effect of quercetin on endothelial cells injured by hypoxia and reoxygenation. Front Pharmacol. (2021) 12:732874. 10.3389/fphar.2021.732874 34744717 PMC8564287

[162] Conquer JA Maiani G Azzini E Raguzzini A Holub BJ. Supplementation with quercetin markedly increases plasma quercetin concentration without effect on selected risk factors for heart disease in healthy subjects. J Nutr. (1998) 128(3):593–7. 10.1093/jn/128.3.593 9482769

[163] Stone NJ Robinson JG Lichtenstein AH Bairey Merz CN Blum CB Eckel RH 2013 ACC/AHA guideline on the treatment of blood cholesterol to reduce atherosclerotic cardiovascular risk in adults: a report of the American college of cardiology/American heart association task force on practice guidelines. J Am Coll Cardiol. (2014) 63(25 Pt B):2889–934. 10.1016/j.jacc.2013.11.002 24239923

[164] Hansson GK. Inflammation, atherosclerosis, and coronary artery disease. N Engl J Med. (2005) 352(16):1685–95. 10.1056/NEJMra043430 15843671

[165] Cai H Harrison DG. Endothelial dysfunction in cardiovascular diseases: the role of oxidant stress. Circ Res. (2000) 87(10):840–4. 10.1161/01.RES.87.10.840 11073878

[166] Mihaylova B Emberson J Blackwell L Keech A Simes J Barnes EH The effects of lowering LDL cholesterol with statin therapy in people at low risk of vascular disease: meta-analysis of individual data from 27 randomised trials. Lancet. (2012) 380(9841):581–90. 10.1016/S0140-6736(12)60367-5 22607822 PMC3437972

[167] Sirtori CR Galli C Anderson JW Sirtori E Arnoldi A. Functional foods for dyslipidaemia and cardiovascular risk prevention. Nutr Res Rev. (2009) 22(2):244–61. 10.1017/S0954422409990187 20003590

[168] Oh WY Ambigaipalan P Shahidi F. Quercetin and its ester derivatives inhibit oxidation of food, LDL and DNA. Food Chem. (2021) 364:130394. 10.1016/j.foodchem.2021.130394 34167006

[169] Janisch KM Williamson G Needs P Plumb GW. Properties of quercetin conjugates: modulation of LDL oxidation and binding to human serum albumin. Free Radic Res. (2004) 38(8):877–84. 10.1080/10715760410001728415 15493462

[170] Gnoni GV Paglialonga G Siculella L. Quercetin inhibits fatty acid and triacylglycerol synthesis in rat-liver cells. Eur J Clin Invest. (2009) 39(9):761–8. 10.1111/j.1365-2362.2009.02167.x 19508303

[171] Sahebkar A. Effects of quercetin supplementation on lipid profile: a systematic review and meta-analysis of randomized controlled trials. Crit Rev Food Sci Nutr. (2017) 57(4):666–76. 10.1080/10408398.2014.948609 25897620

[172] Massi A Bortolini O Ragno D Bernardi T Sacchetti G Tacchini M Research progress in the modification of quercetin leading to anticancer agents. Molecules. (2017) 22(8):1270. 10.3390/molecules22081270 28758919 PMC6152094

